# Efficacy and prognostic analysis of bronchoscopic intervention in elderly patients with tracheobronchial tuberculosis

**DOI:** 10.3389/fmed.2025.1624985

**Published:** 2026-01-05

**Authors:** Yueyang Tian, Tianhe Su, Leilei Shen, Jikun Zhou, Yang Sun

**Affiliations:** 1 Department of Clinical Laboratory Medicine, The Fifth Hospital of Shijiazhuang, Shijiazhuang, Hebei, China; 2 Department of Tuberculosis, The Fifth Hospital of Shijiazhuang, Shijiazhuang, Hebei, China; 3 The Institute of Medical Research, The Fifth Hospital of Shijiazhuang, Shijiazhuang, Hebei, China

**Keywords:** tracheobronchial tuberculosis, elderly patients, bronchoscopic intervention, airway narrowing, prognostic analysis

## Abstract

This study investigated the efficacy and prognosis of bronchoscopic interventional therapy in elderly patients with tracheobronchial tuberculosis (TBTB). We prospectively included 142 elderly patients with TBTB for interventional treatments such as bronchoscopic balloon dilation, mechanical evacuation, and stent implantation, and long-term follow-up (median 28.4 months). The results showed that the technical success rate of interventional therapy was 90.8, 92.3% of patients had improved symptoms, and FEV1 was significantly improved from 43.2% at baseline to 65.8% after surgery (*p* < 0.001). Fibrostenosis TBTB (OR 3.42), Freitag grade 5 stenosis (OR 2.76), stenosis length > 2 cm (OR 2.18), and Charlson comorbidity index ≥3 (OR 1.98) were independent predictors of poor prognosis. Restenosis occurred in 27.1% of patients after surgery, and the survival rate of older patients (≥75 years) was significantly lower than that of younger patients (81.6% vs. 94.2%, *p* = 0.003). In this study, we propose a prognostic risk scoring model and confirm that bronchoscopic intervention is safe and effective in elderly patients with TBTB, but patient selection is crucial.

## 1 Introduction

Tracheobronchial tuberculosis (TBTB) is a disease of public health concern, especially for older adults, as the condition’s diagnosis and treatment come with difficulties (1). This form of tuberculosis, which infects the tracheobronchial tree, occurs in 10–40% of patients suffering from active pulmonary tuberculosis and is particularly morbid and mortal (2). In the context of the elderly, typically characterised as persons aged 65 years and older, there exists a unique pathophysiological response due to immunosenescence, multi-morbidity, and age-related changes in anatomy, which makes the presentation and management of the illness complex (3).

The symptoms of TBTB in older adults are far more pronounced and deviant than in younger populations, presenting TBTB with the challenge of being diagnosed late. Patients of advanced ages with TBTB often present with non-specific signs like histopathologic chronic cough, dyspnoea, or recurrent infections which are specific to the lungs but are frequent at this age range. Eventually, airway stenosis becomes a critical complication as it progresses with the disease, causing significant damage to the functional abilities and quality of life of patients. About 68% of patients suffering from TBTB have, in conjunction with sufficient anti-tubercular medication, the development of bronchial stenosis, along with significant proportions of advanced cases that are severe stenosis which require some form of interventional techniques.

Recent studies have highlighted the growing burden of TBTB in ageing populations. A 2024 multicenter cohort from China (4) reported that a high disease density of 1,292.5 cases per 100,000 person-years amongst older adults with latent tuberculosis infection (LTBI), highlighting the urgent need for targeted prevention and control strategies in this vulnerable population. Similarly, a 2025 study in Xinjiang (5), China, found that amongst treatment-naive PTB patients, 43.4% developed TOPD during a one-year follow-up, with age, BMI, ESR, and symptom duration identified as significant risk factors. A predictive model using these factors demonstrated 89% sensitivity and 83% specificity, underscoring the need for early detection and tailored interventions to improve patient outcomes.

Despite advances in bronchoscopic techniques, long-term functional outcomes and the role of airway remodelling in elderly TBTB remain poorly understood. A 2024 systematic review (6) emphasised the lack of longitudinal lung function data in elderly TBTB patients, particularly regarding the impact of restenosis on FEV1 trajectories. Most studies rely on cross-sectional comparisons, which may misinterpret natural fluctuations as treatment failure.

Bronchoscopic intervention can be regarded as a useful therapeutic approach for TBTB-induced airway stenosis which may assist in alleviating symptoms, improving lung function, and even influencing the course of the disease. These interventions encompass a range of techniques such as balloon dilation, cryotherapy, stenting, and laser therapy which have different mechanisms of action and their own specific use cases each (7). There is an increasing body of literature documenting the effectiveness of these interventions amongst the general adult population, but literature addressing the uses and outcomes amongst older patients is limited and sparse.

The treatment of older individuals with tuberculosis (TB) presents unique complications, particularly due to their low physiological reserve, possible contraindications from other comorbid conditions, altered pharmacokinetics of adjunctive medications, and decreased tolerance for procedures. Therefore, any evaluation concerning the efficacy, safety, and prognostic value of bronchoscopic interventions in this age cohort requires detailed study to inform clinical practise and enhance outcomes. This study focuses on examining the therapeutic effectiveness, safety issues, outcome determinants, and their predictive modelling of clinical decisions in this vulnerable population. This study addresses this gap by prospectively evaluating the long-term efficacy, safety, and lung function trajectories in elderly TBTB patients, with a focus on the mechanistic role of restenosis using longitudinal mixed-effects modelling.

## 2 Materials and methods

### 2.1 Study design and participants

The cohort study of the participants was performed from January 2018 to December 2023 and was completed at the Department of Respiratory Medicine of a University Hospital Tertiary Care Centre. The hospital’s institutional review board granted permission for the protocol, and informed consent was not sought from patients when retrospectively analysing the data.

Included in the study were patients aged 65 years and older diagnosed with tracheobronchial tuberculosis (TBTB) based on one of the following criteria: (1) positive culture of *Mycobacterium tuberculosis* from bronchial washing; (2) bronchial biopsy showing granulomatous inflammation with caseous necrosis; or (3) TBTB findings at bronchoscopy in a patient with pulmonary tuberculosis who had a positive culture for pulmonary tuberculosis. Healthcare providers performing endoscopic interventions on the air passages prior to advanced nursing systems for ventilatory dependent patients treated with bronchial techniques after splitting the airway were included. They all developed marked airway stenosis, defined as a 50% or greater reduction of the TBTB airway lumen, subsequent to TBTB, and underwent at least one bronchoscopy.

Patients were excluded if they had: (1) a malignant tumour causing obstruction to an airway; (2) stenosis of the airway as a result of other granulomatous diseases; (3) a previous operation for narrowing of the airway; (4) active bleeding from the lungs; (5) chronic heart and lung failure which made bronchoscopy infeasible; and (6) lack of complete documents or medical history and monitoring records.

The data gathered included age, sex, smoking details, the individual’s place of residence, and their BMI. Clinical factors included the duration of symptoms prior to intervention, tuberculosis treatment history, comorbidities (using the Charlson Comorbidity Index), baseline functional status (using the Eastern Cooperative Oncology Group performance status scale), and the presenting symptoms of the disease. These parameters also included complete blood count, albumin levels, renal and liver activity, erythrocyte sedimentation rate, and C-reactive protein. Pulmonary function tests were completed 2 weeks prior to the intervention and included FEV1, FVC, and FEV1/FVC ratio.

Patients received standard anti-tuberculosis medication prior to, during, and following the bronchoscopic intervention, as per the national guidelines. The first 2 months consisted of an intensive phase, which included a four-drug combination of isoniazid, rifampicin, ethambutol, and pyrazinamide, followed by a continuation phase of isoniazid and rifampicin. The treating physician dictated the duration of the treatment—at least 6 months—in accordance with the clinical, radiological, and microbiological responses.

The sample size was determined based on the first outcome, which is improvement in a patient’s condition from the clinical perspective after performing the bronchoscopic intervention. Considering a clinical improvement proportion of 70% from literature in general adult populations and assuming an 8% margin of error and 95% confidence level, the sample size was determined to be at least 126 patients. Considering possible attrition or missing data, our goal was to enrol a minimum of 140 patients for the final analysis.

### 2.2 Diagnostic criteria and evaluation methods

**Figure 1:**
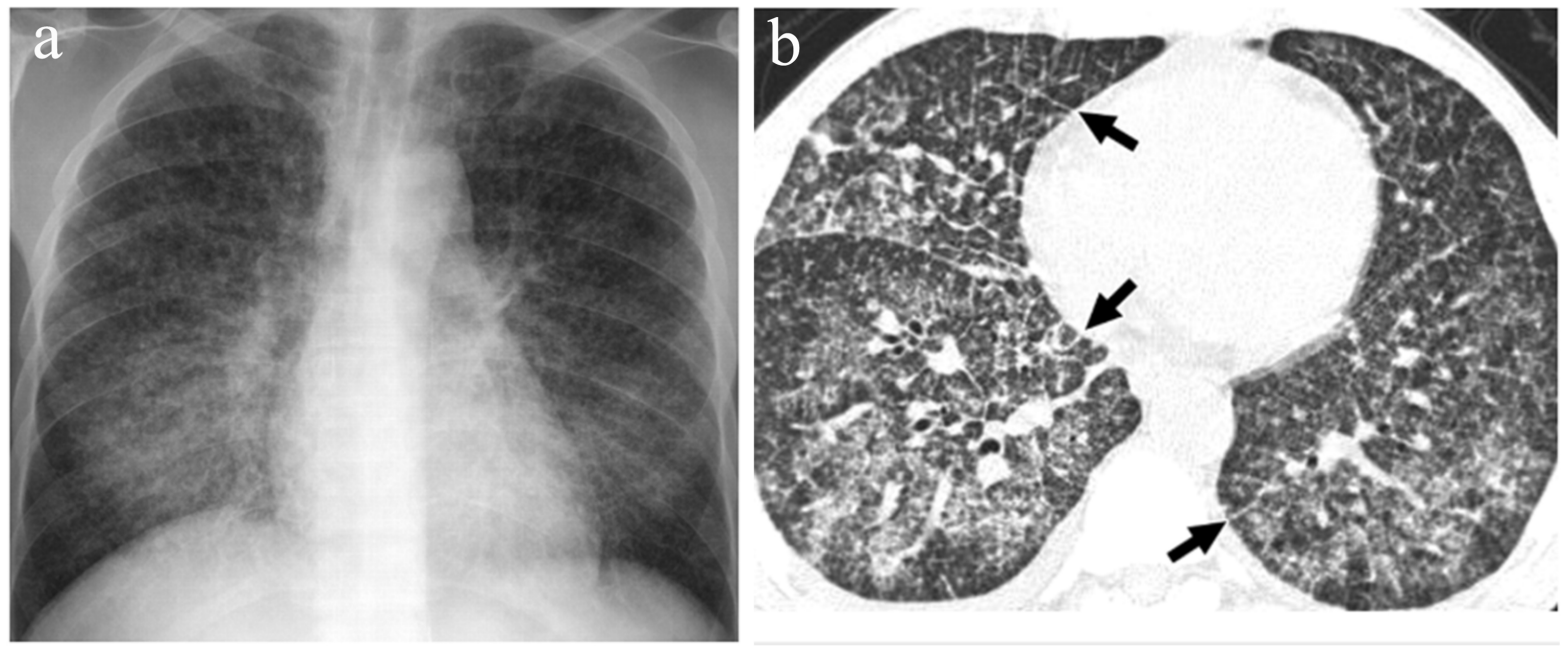
Imaging findings of tracheobronchial tuberculosis. **(a)** Chest X-ray for a man suffering from the tracheobronchial form of tuberculosis. Chest X-rays of patients with tracheobronchial tuberculosis may show only subtle changes; “definite” airway lesions are absent, therefore, yielding a low diagnostic sensitivity of routine X-rays. **(b)** High-resolution CT scan demonstrates small nodules and “tree bud signs” most appropriately seen within the lung parenchyma, bronchiogenic spread. These changes are often difficult to demonstrate with conventional X-rays. With HRCT, the intrinsic nature, scope and severity of lesions have the accuracy which is significant for the early diagnosis of TBTB.

The diagnosis of TBTB relies on a synthesis of clinical, radiologic, microbiologic and histopathologic workup (8). All patients completed chest x-ray and HRCT scanning for evaluation of involvement of the tracheobronchial tree and lung parenchyma. TBTB has a low sensitivity with chest x-ray, and some patients may demonstrate features of minimal abnormality with subtle airway lesions, as depicted in Figure 1a (9).

CT scans can be more informative for the diagnosis of TBTB since they demonstrate small lesions which are harder to find using conventional x-ray machines. Extensive changes and the insertions of two black arrows indicating the tiny nodules in the lung parenchyma are quite visible in the high-resolution CT image (Figure 1b). Lee et al. demonstrated that active tuberculosis on HRCT included oedema and irregular bronchial wall changes, while fibrotic changes only incorporated smooth edges with no significant airway wall thickening and oedema, with lesions (9). The visibility of the defaults and extent of tracheobronchial involvement, including the “tree bud sign” indicative of bronchogenic dissemination, are shown more clearly by HRCT scan, as demonstrated in Figure 1b.

**Figure 2:**
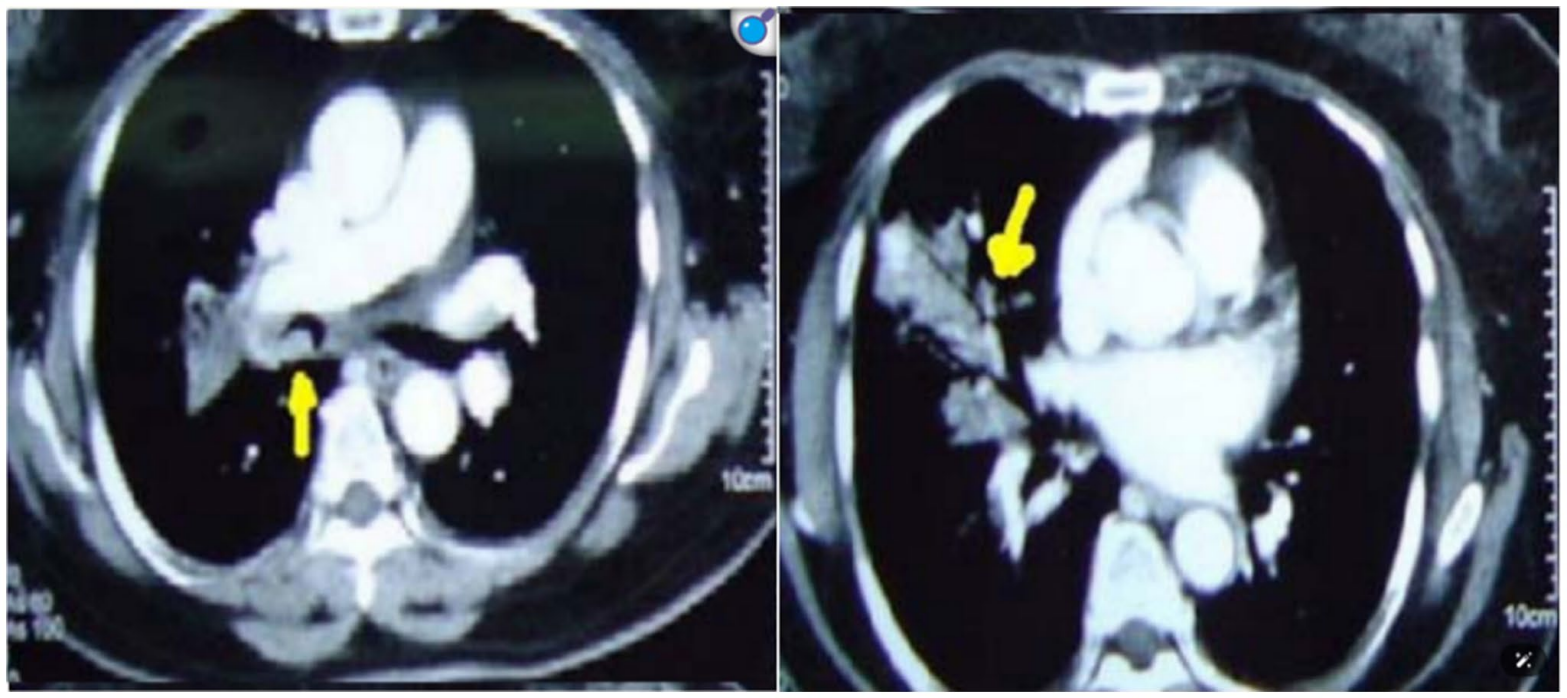
Chest CT image of a patient with tracheobronchial tuberculosis.

As previously mentioned, the detection of small lesions that do not appear on conventional X-rays is of great importance in the early diagnosis of TBTB. In Figure 2, the area pointed out by the yellow arrow exhibits bronchial wall thickening and lumen stenosis which is characteristic of TBTB. In comparison with obstructions, Lee et al. demonstrated that active HRCT features of tuberculosis are oedema and irregularity of the bronchial wall and fibrotic lesions are smooth, without significant airway wall thickening or oedema leading to fine structures (9).

The region pointed out by the yellow arrow demonstrates bronchial wall thickening and lumen narrowing. High-resolution CT (Computed Tomography) can demonstrate changes in airway walls and stenosis of the lumen, which is very important for assessing the degree and nature of TBTB lesions.

**Figure 3:**
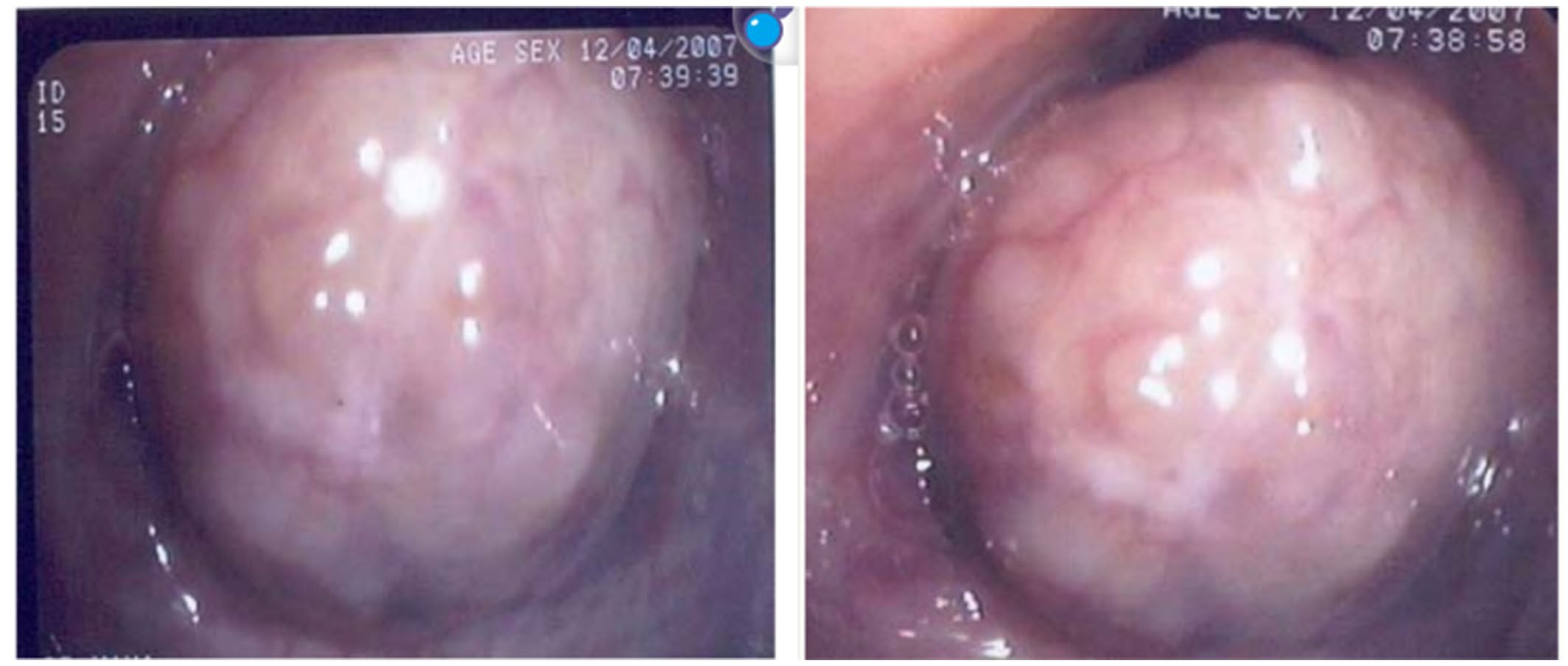
Bronchoscopic image of tracheobronchial tuberculosis.

Bronchoscopy is the major procedure in the work-up of TBTB. All patients have had flexible bronchoscopy performed (BF-1 T260, Olympus, Tokyo, Japan) under local anaesthesia and conscious sedation. Figure 3 illustrates the typical bronchoscopic features of TBTB with tumour and granulation-like hyperplastic changes causing bronchial lumen stenosis. In the opinion of Chung et al., TBTB can be divided into seven subtypes of: active caseous, edematous, fibrostenosis, neoplastic, granulogenic, ulcerative, and nonspecific bronchitis (2). The lesions illustrated in Figure 3 pertain to the neoplastic type of TBTB, which has a poor prognosis; they mostly progress to fibrostenosis in 3 months.

Pictures demonstrate endobronchial tumour-like or granuloid changes causing marked bronchial lumen stenosis. This is in accordance with Chung’s description of neoplastic TBTB that is often accompanied by unfavourable clinical outcomes and fibrostensosis complications during treatment.

Using the bronchoscopic stenosis grading system suggested by Freitag et al., airway stenosis is subdivided into five grades: grade 1 (obstruction <25%), grade 2 (obstruction 26–50%), grade 3 (obstruction 51–75%), grade 4 (obstruction 76–90%), and grade 5 (obstruction >90%) (10, 11). Bronchial lavage samples from the affected region are taken during bronchoscopy for AFB smear, mycobacterial culture, and GeneXpert MTB/RIF assay, and bronchoscopy is complemented by bronchial biopsy from visible lesions or stenotic areas for histopathology and culture.

Respiratory capabilities were powered using the instructions and protocols set forth by the American Thoracic Society and European Respiratory Association’s standards on pulmonary functions (ATS/ERS) (12). Values that were recorded included forced vital capacity (FVC), forced expiratory volume in 1 s (FEV1), FEV1/FVC ratio, and peak expiratory flow (PEFR). These measurements were done at baseline (within 2 weeks before the intervention), 1 month, 3 months, 6 months, and then every subsequent 6 months after the intervention. The PEFR and FVC parameters were obtained in absolute terms and as percentages based on age, sex, height and ethnicity activated.

Disabling health conditions with mMRC Grade 0 to 4 (dyspnoea only during strenuous exercise) were evaluated and assessed with the St. George’s Respiratory Questionnaire (SGRQ) which determines degradation of health issues with emphysema classified into three categories: symptoms, activity, and impact. Higher scores indicate worse health status, the minimum clinically important difference is defined as 4 units.

Monitoring of tuberculosis activity was done by clinical assessment, AFB serial sputum examination, and chest X-ray. Complete response to anti-tuberculous therapy comprises absolute resolution of symptoms, negative sputum culture of *Mycobacterium tuberculosis*, and radiographic amelioration.

Patients with chronic heart failure (NYHA class III–IV) or advanced chronic obstructive pulmonary disease (GOLD stage III–IV) were excluded to ensure clinical homogeneity and reduce confounding in the assessment of restenosis. These conditions can independently cause dyspnea and airway collapse, making it difficult to attribute symptoms or imaging changes solely to TBTB-related stenosis.

### 2.3 Bronchoscopic intervention protocol

Bronchoscopic interventional treatment is performed in an interventional bronchoscopy room specially equipped with fluoroscopic guidance capabilities and advanced monitoring facilities. All surgeries are performed by interventional pulmonologists with at least 5 years of experience in therapeutic bronchoscopy. Patients are evaluated by a multidisciplinary team that includes pulmonologists, thoracic surgeons, anesthesiologists, and infectious disease specialists to determine the best treatment option prior to interventional therapy.

The choice of specific bronchoscopic technique is based on the morphological features of the stenosis, its location, extent, texture (fibrous versus inflammatory), and the patient’s clinical status. For procedures requiring deep sedation or general anaesthesia, a rigid bronchoscope (Karl Storz, Tuttlingen, Germany) is used as the primary platform, with a flexible bronchoscope introduced through a rigid tube for distal intervention if necessary. For simpler procedures, a therapeutic flexible bronchoscope (BF-1 T180 or BF-1TQ190, Olympus, Tokyo, Japan) is used (13).

**Figure 4:**
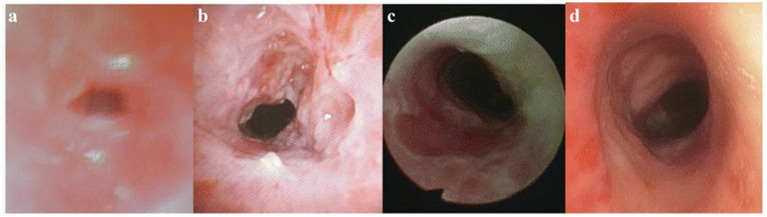
Bronchoscopic interventional treatment of tracheobronchial tuberculosis. **(a)** Airway patency after balloon dilation. **(b)** Residual stenosis post-dilation. **(c)** Airway during further intervention. **(d)** Technical success (residual stenosis <50%).

The CRE balloon dilator (Boston Scientific, Natick, MA, United States) with a balloon diameter of 6 to 18 mm is used to perform balloon bronchoplasty. The balloon is placed in the stenosis under fluoroscopic or direct vision guidance. Stepwise inflation is done to achieve the target pressure of 3–6 atm and held for 60–90 s. Successive pressures can also be applied to achieve dilation at the required rate (staged inflation). This method has been demonstrated to successfully relieve narrowing of the airways due to tuberculosis, as seen in Figure 4a, and what can appear as severe stenosis before treatment (Figure 4d) regains good patency after the successful intervention. Lesions that are volumetric and encroach upon the airway cavity are surgically removed by using clamps, rigid bronchoscopy, or operative curettage (14).

Electrocautery, utilising an electrosurgical unit like the ERBE VIO 300D (Tübingen, Germany), is performed for patients with fibrous stenosis who do not respond to balloon dilation. Cutting and coagulation are performed based on the tissue characteristics. For hypervascular mucosal lesions and other interventional procedures, argon plasma coagulation (ERBE APC 2, Tübingen, Germany) is employed and the power settings for haemostasis is adjusted between 25 and 40 watts. In more selective cases, thick fibrous stenosis is treated with neodymium-doped yttrium aluminium garnet (nd-YAG) lasers (Lumenis, Santa Clara, CA, United States) set at 15–40 watts in pulsed mode (15). There is also some residual stenosis in the airway lumen after initial dilation which may require further intervention as shown in Figure 4b and the state during further treatment is illustrated in Figure 4c.

Nitrous oxide is used as the cryoagent, and a flexible cryoprobe (ERBE, Tübingen, Germany) is employed to perform cryotherapy at approximately −80 °C. The freeze–thaw cycle for each site consists of 20 to 30 s of freezing followed by natural thawing, which is done 2–3 times for each site. In patients with greater than 50% residual stenosis after these techniques are employed or in the case of patients with a high suspicion of restenosis, a silicone stent (Dumon stent, Novatech, plan de Grasse, France) may be placed or a self-expanding metal stent (Ultraflex, Boston Scientific, Natick, MA, United States) placed. The stent size is selected based on measurement of the normal airway at the proximal and distal end of the stenosis and overly set at 10–20% in diameter to ensure secure holding.

Throughout the entire procedure, vital signs, oxygen saturation, end-tidal carbon dioxide and electrocardiogram are continually monitored. Oxygen supplementation is given to maintain a saturation of more than 90%. To minimise postoperative oedema, intravascular corticosteroids (methylprednisolone 40–80 mg) are administered prior to intervention. If required, topical application of epinephrine (1:10,000 solution) will be used to stop bleeding. Technical success is described as accomplishing airway patency while the residual stenosis is less than 50% of the normal cavity diameter as evaluated by bronchoscopy immediately after intervention, as shown in Figure 4d. Achievement of functional success is described by at least one level improvement on the MRC dyspnoea scale mMRC. And/or FEV1 shows improvement of at least 15% from baseline value. In the interventional therapy figures for d, a complete interventional treatment process is depicted alongside the resultant change observed from severe stenosis to successful dilation, effectively illustrating the outcome achieved through interventional therapy.

### 2.4 Follow-up protocol and outcome assessment

Each patient was routinely scheduled and followed up on for treatment response and possible complications. They were first evaluated clinically, with imaging, pulmonary function tests, and if indicated, bronchoscopy at one, three-, and six-months post-intervention, then biannually thereafter. All workup was completed at each follow-up visit (16).

Clinical evaluation comprised signs and symptoms, and physical examination. Levels of dyspnoea were assessed using the modified Medical Research Council (mMRC) dyspnoea scale, which ranges from 0 (only breathless with strenuous exercise) to 4 (breathless upon dressing/undressing). At the same time, cough was assessed using a visual analogue scale (VAS), 0 representing no cough and 10 the most severe cough imaginable (17).

**Figure 5:**
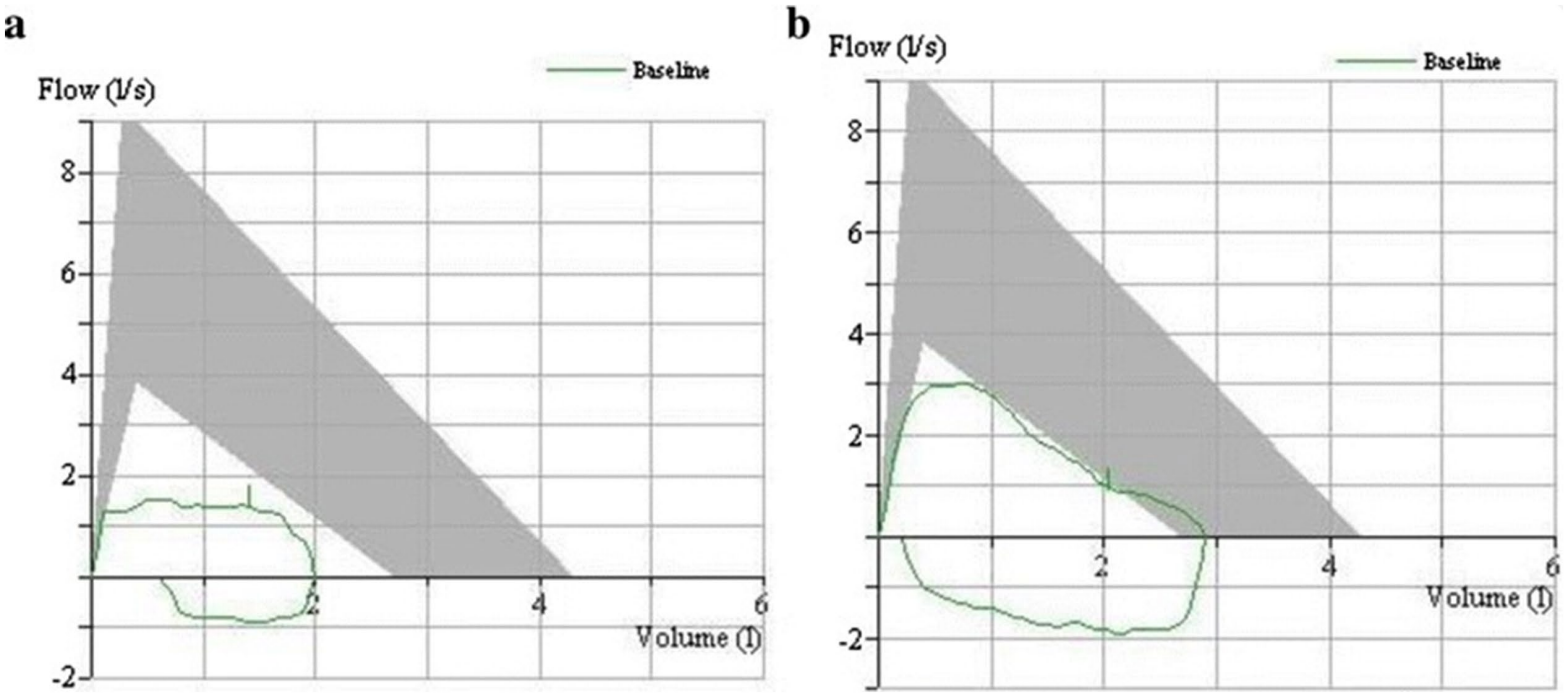
Flow–volume loops before and after intervention. **(a)** Pre-intervention flow–volume loop. **(b)** Post-intervention flow–volume loop.

Objective indicators of intervention efficacy were evaluated using pulmonary function testing. This included measuring forced vital capacity (FVC), forced expiratory volume in one second (FEV1), and the FEV1/FVC ratio as well as peak expiratory flow rate (PEFR) measurements. Moreover, assessing the degree and location of airway stenosis through flow-volume loops was especially insightful (18). As it is illustrated in Figure 5, the flattening of both the inspiratory and expiratory limbs of the flow-volume loop indicates fixed airway stenosis due to tracheobronchial tuberculosis, which is known to be a sensitive indicator for evaluating treatment efficacy. Figure 5a shows the pre-treatment flow-volume loop where the patient data depicted in green (5a) is significantly outside of the normal reference range depicted as the grey area; this demonstrates marked airflow limitation. Figure 5b shows post-treatment improvement in the flow-volume loop, although it is still not fully within the normal range.

Investigations included radiography and where appropriate chest CT, to assess the extent of parenchymal lesions, airway stenosis, and secondary complications like obstructive pneumonia or atelectasis. With patients undergoing stent placement, these studies also evaluated stent position and patency.

For participants receiving balloon dilatation or stenting, treatment success encompassed: (1) marked improvement in symptoms with at least a one-grade reduction on the mMRC scale; (2) an increase in FEV1 of at least 15% from the baseline value; and (3) airway constriction reversal of no less than 50% as determined by bronchoscopy (19). In cases of restenosis identified during follow-up, repeat intervention may be required. Restenosis was defined within the context of symptom recurrence along with a decline on the flow–volume loop examination and/or bronchoscopic confirmation of greater than 50% reduction in airflow through the airway.

Evaluation of the long-term outcomes included: (1) time with no associated symptoms; (2) time free of restenosis; (3) rate of subsequent interventions; and (4) long-term changes in pulmonary function parameters. Safety assessment included complications related to the intervention such as bleeding, pneumothorax, rupture of bronchus, migration of the stent, and formation of granulation tissue (20).

### 2.5 Subgroup analysis of interventional strategies in fibrostenotic TBTB patients

Given the heterogeneity of interventional bronchoscopic techniques used in the management of tracheobronchial tuberculosis (TBTB), we conducted a predefined subgroup analysis focusing on patients with fibrostenotic disease — a phenotype at high risk for restenosis. The objective was to evaluate whether specific interventions, particularly stent placement, were independently associated with reduced restenosis risk after adjusting for potential confounders.

Within the fibrostenotic subgroup, patients were categorised based on whether they received airway stent placement during their treatment course. Restenosis-free survival was compared between the stent and non-stent groups using Kaplan–Meier estimation and the log-rank test. To assess the independent effect of stent placement, a multivariable Cox proportional hazards regression model was fitted, adjusting for the following covariates: age, Charlson Comorbidity Index, baseline stenosis length (in cm), Freitag grade, total procedure duration (in minutes), and the use of concomitant interventions (balloon dilatation, laser therapy, mechanical debridement, and argon plasma coagulation).

The primary outcome was time to restenosis, defined as the interval from the first interventional procedure to the radiological or bronchoscopic confirmation of recurrent airway narrowing requiring retreatment. Patients without restenosis were censored at the date of last follow-up. The proportional hazards assumption was evaluated using log–log survival plots and Schoenfeld residuals. All analyses were performed using Python (v3.11) with the lifelines package (version 0.27.0). A two-sided *p*-value <0.05 was considered statistically significant.

### 2.6 Longitudinal analysis of lung function

To evaluate the trajectory of FEV1 over time and the impact of restenosis, we performed a linear mixed model (LMM) with random intercepts and slopes for time. The model included fixed effects for time (in months), restenosis status, and their interaction, adjusted for age, Charlson comorbidity index, stenosis length, Freitag grade, and procedure duration. This approach accounts for within-subject correlation and allows assessment of whether restenosis accelerates FEV1 decline.

### 2.7 Statistical analysis methods

Statistical analyses were performed using Python (v3.11; Python Software Foundation) with the following packages: pandas (v2.0.3) for data manipulation, statsmodels (v0.14.0) for linear mixed models, lifelines (v0.27.0) for survival analysis, and matplotlib (v3.7.2) and seaborn (v0.12.2) for visualisation. Additional analyses were verified using R (v4.1.2; R Foundation for Statistical Computing) and SPSS (v26.0; IBM Corp., Armonk, NY, United States). The sample size was determined from the primary endpoint of clinical improvement after bronchoscopic intervention. Assuming a clinical improvement rate of 70% based on previous studies in adult populations, along with an 8% margin of error at a 95% confidence level, the minimum required sample size was determined to be 126 patients (2). In order to account for missing data or attrition, we set out to include a minimum of 140 patients in the study.

For continuous variables that were normally distributed, data was represented as mean ± standard deviation (SD) while non-normally distributed data was expressed as median with interquartile range (IQR). The Shapiro–Wilk test was utilised to evaluate the normality of the data. Frequencies and percentages were used to display categorical variables. For the demographic and baseline clinical characteristics, group comparisons were conducted with the student’s *t*-test or Mann Whitney U test for continuous variables, and chi-square or Fisher’s exact test for categorical ones as it suited best.

Pulmonary intervention metrics such as FEV1, FVC, FEV1/FVC, and PEFR were analysed for pre- and post-intervention difference using paired *t*-tests or Wilcoxon signed-rank tests based on data distribution. To assess changes in these parameters over multiple time points during postoperative follow-up, repeated measures ANOVA or Friedman’s test was performed. For categorical variables like the presence of certain bronchoscopic findings before and after intervention, McNemar test was applied for comparison (21).

Kaplan–Meier survival curves were created for time-to-event analyses to estimate event-free survival rates such as restenosis-free and re-intervention-free survival. Survival distribution between groups was compared using log-rank test for paired analyses. Identification of factors related to the risk of restenosis and intervention failure was performed using Cox proportional hazard regression models. Prior to testing, the proportional hazards assumption was validated using Schoenfeld residuals (22).

The presence of technical and functional success was assessed using multivariate logistic regression, with the outcome presented as odds ratios (OR) with corresponding 95% confidence intervals (CI). Univariate analysis with a *p* value < 0.10 was used to set the independence criteria for multivariate model variables. To evaluate the presence of multicollinearity amongst predictor variables, variance inflation factors (VIF) greater than 5 were used.

A propensity score matching analysis was performed to eliminate prospective bias in treatment selection. The nearest neighbour 1:1 method was used for matching with a calliper width of 0.2 for the standard deviation of the logit of the propensity score. Standardised mean differences were computed for the matched groups to evaluate the balance of covariates, with less than 0.1 indicating a favourable balance (23).

All analyses were conducted under the assumption that two-sided *p* values of less than 0.05 would be significant. Where applicable, the Bonferroni correction was applied to control for multiple comparisons. Other than that, missing data were dealt with using sophisticated techniques based on the assumption that data were missing randomly. Various sensitivity analyses were carried out to evaluate the validity of the findings under different assumptions regarding the missing data.

Descriptive statistics were used to summarise baseline characteristics. Categorical variables are presented as frequencies and percentages, while continuous variables are expressed as mean ± standard deviation or median (interquartile range), as appropriate.

The primary outcome was time to restenosis, defined as the interval from the first interventional bronchoscopy to the diagnosis of restenosis on follow-up bronchoscopy or imaging. Patients who did not experience restenosis were censored at the date of last follow-up.

Kaplan–Meier survival curves were constructed to estimate restenosis-free survival, and differences between groups were assessed using the log-rank test. A multivariable Cox proportional hazards regression model was fitted to evaluate the independent association between stent placement and restenosis risk in fibrostenotic patients, adjusting for potential confounders including age, Charlson Comorbidity Index, stenosis length, Freitag grade, procedure duration, and concomitant interventions (balloon dilatation, laser therapy, mechanical debridement, and argon plasma coagulation).

Proportional hazards assumptions were visually inspected using log–log survival plots. The model’s discriminative performance was assessed using Harrell’s concordance index (C-index). All statistical analyses were performed using Python (v3.11) with the pandas, scipy, and lifelines packages. A two-sided *p*-value < 0.05 was considered statistically significant.

## 3 Results

### 3.1 Baseline characteristics of the study population

One hundred forty-two elderly patients with tracheobronchial tuberculosis (TBTB) and a history of undergoing bronchoscopic intervention were included in the final analysis. The cohort comprised 87 (61.3%) females and 55 (38.7%) males, with a mean age of 68.7 ± 6.2 years (range, 65–89 years). The median time between the diagnosis of TBTB and commencement of bronchoscopic intervention was 5.2 months (IQR, 3.4–8.7 months). Demographic and clinical characteristics of the study population are presented in Table 1.

**Table 1:** Demographic and clinical characteristics of elderly patients with tracheobronchial tuberculosis (*N* = 142).

Characteristic	Value
Demographic characteristics	
Age (years), mean ± SD	68.7 ± 6.2
Age range (years)	65–89
Gender, *n* (%)	
Female	87 (61.3%)
Male	55 (38.7%)
Time between TBTB diagnosis and intervention (months), median (IQR)	5.2 (3.4–8.7)
Clinical characteristics	
Presenting symptoms, *n* (%)	
Dyspnea	121 (85.2%)
Chronic cough	107 (75.4%)
Stridor	68 (47.9%)
Hemoptysis	23 (16.2%)
mMRC dyspnea score at presentation, median (IQR)	3 (2–4)
Prior anti-tuberculosis therapy, *n* (%)	98 (69.0%)
Duration of prior anti-TB therapy (months), median (IQR)	6.5 (4.2–9.8)
Multidrug-resistant tuberculosis, *n* (%)	22 (15.5%)
Comorbidities, *n* (%)	
Hypertension	89 (62.7%)
Diabetes mellitus	63 (44.4%)
Chronic obstructive pulmonary disease	51 (35.9%)
Charlson comorbidity index, median (IQR)	2 (1–4)
Lesion Location, *n* (%)	
Left main bronchus	58 (40.8%)
Right main bronchus	43 (30.3%)
Trachea	31 (21.8%)
Multiple locations	10 (7.0%)
Chung classification, *n* (%)	
Fibrostenotic type	76 (53.5%)
Edematous-hyperemic type	28 (19.7%)
Actively caseating type	21 (14.8%)
Tumorous type	10 (7.0%)
Granular type	7 (4.9%)
Freitag grading system, *n* (%)	
Grade 3 (51–75% obstruction)	53 (37.3%)
Grade 4 (76–90% obstruction)	61 (43.0%)
Grade 5 (>90% obstruction)	28 (19.7%)
Baseline pulmonary function	
FEV1 (L), mean ± SD	1.21 ± 0.43
FEV1 (% predicted), mean ± SD	43.2 ± 15.6
FEV1/FVC ratio, mean ± SD	0.58 ± 0.14
Fixed upper airway obstruction patterns, *n* (%)	103 (72.5%)
Minimum luminal diameter at stenotic segment on CT (mm), mean ± SD	3.5 ± 1.6

IQR, interquartile range; TBTB, tracheobronchial tuberculosis; mMRC, modified Medical Research Council; FEV1, forced expiratory volume in 1 s; FVC, forced vital capacity; CT, computed tomography.

The most common presenting feature in our cohort was dyspnoea ( n =121, 85.2%), followed by chronic cough ( n =107, 75.4%), stridor ( n =68, 47.9%), and haemoptysis ( n =23, 16.2%). The median mMRC dyspnoea score at presentation was 3 (IQR, 2–4). Most patients ( n =98, 69.0%) had received prior anti-tuberculosis therapy for a median duration of 6.5 months (IQR, 4.2–9.8 months). Of the 22 (15.5%) patients, multidrug-resistant tuberculosis was documented.

**Figure 6:**
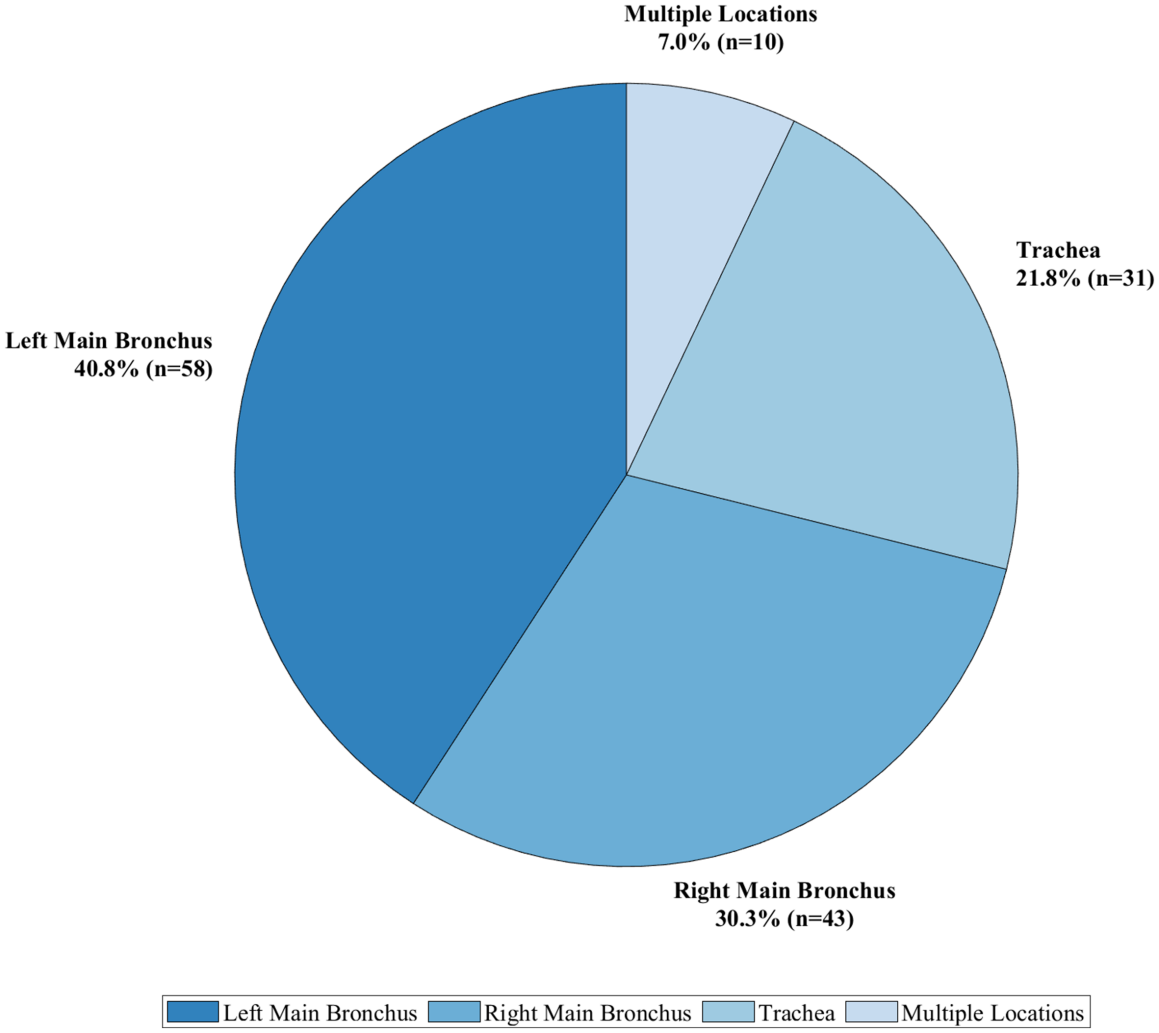
Distribution of lesion locations in elderly patients with tracheobronchial tuberculosis.

Most of the elderly patients in this cohort suffered from chronic comorbidities, the most prevalent being hypertension ( n =89, 62.7%), diabetes mellitus ( n =63, 44.4%), and chronic obstructive pulmonary disease (*n* = 51, 35.9%). The median of the Charlson Comorbidity Index for this group was 2 (IQR, 1–4). According to bronchoscopic examination, the primary sites for the involvement of TBTB lesions were left main bronchus ( n =58, 40.8%), right main bronchus ( n =43, 30.3%), trachea ( n =31, 21.8%) and others ( n =10, 7.0%). The distribution of lesion locations is provided in Figure 6.

**Figure 7:**
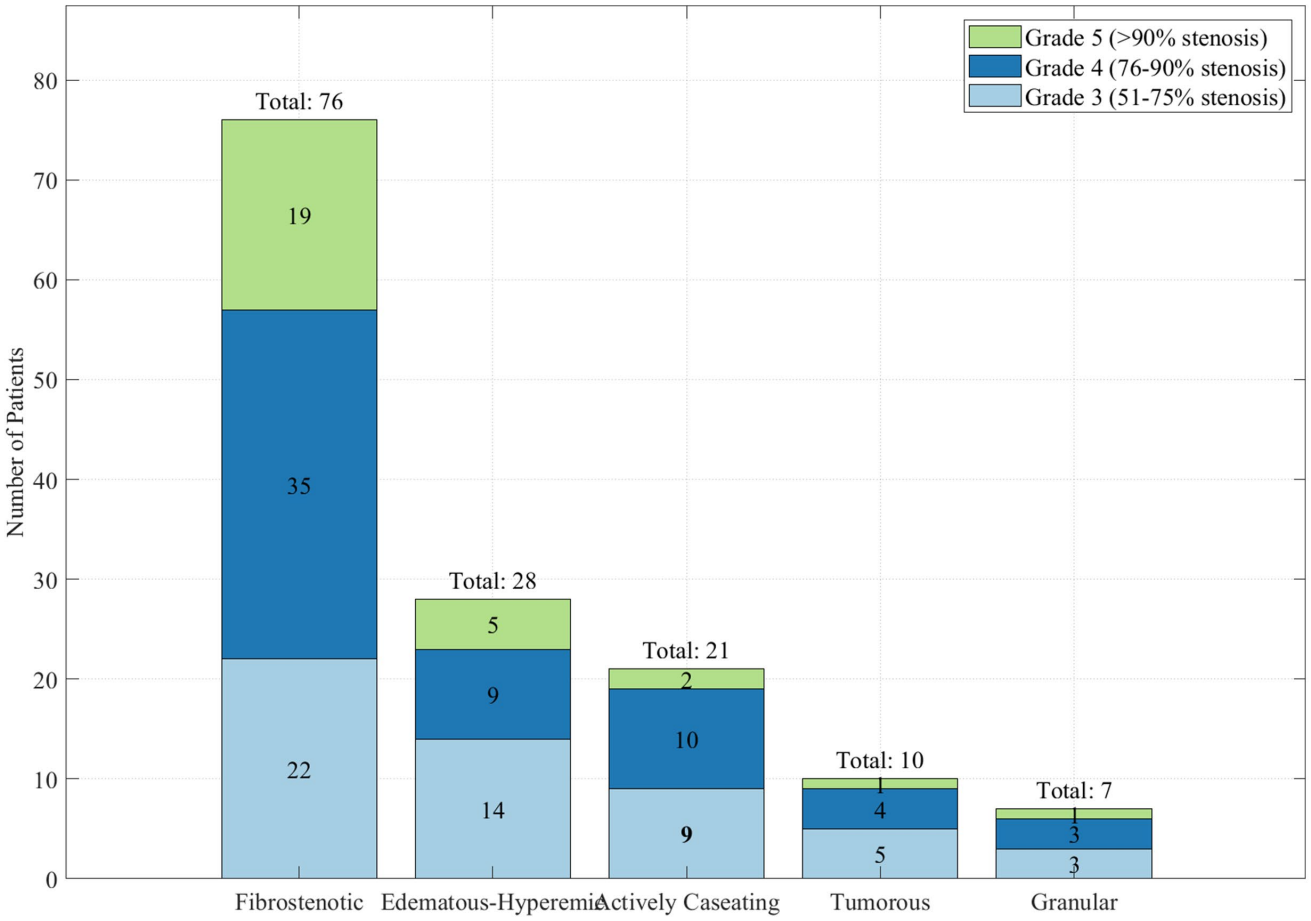
Distribution of Freitag stenosis grades across Chung classifications.

On the basis of Chung classification, fibrostenotic type was the most prevalent ( n =76, 53.5%) followed by edematous-hyperaemic type ( n =28, 19.7%), actively caseating type ( n =21, 14.8%), tumorous type ( n =10, 7.0%) and granular type ( n =7, 4.9%). Using the Freitag grading system, the stenosis was classified as Grade 3 in 53 (37.3%) patients, Grade 4 in 61 (43.0%) patients, and Grade 5 in 28 (19.7%) patients. Freitag grading across various Chung classifications is shown in Figure 7.

Baseline pulmonary function tests showed considerable impairment in airflow, with the mean FEV1 being 1.21 ± 0.43 L (43.2 ± 15.6% of predicted) and a mean FEV1/FVC ratio of 0.58 ± 0.14. Flow-volume loops revealed fixed upper airway obstruction patterns in 103 (72.5%) patients. CT scans showed marked narrowing of the airway with a mean minimum luminal diameter of 3.5 ± 1.6 mm at the stenotic segment.

This pie chart (Figure 6) depicts the anatomical distribution of tracheobronchial tuberculosis lesions in our elderly patient cohort ( N =142). The left main bronchus was the most frequently involved site (40.8%, n =58), followed by the right main bronchus (30.3%, n =43), the trachea (21.8%, n =31), and multiple locations (7.0%, n =10). This distribution pattern demonstrates the predilection of tracheobronchial tuberculosis for the left main bronchus in elderly patients, consistent with previous findings in the literature that suggest anatomical factors and bronchial angulation may influence site predisposition.

This stacked bar chart (Figure 7) illustrates the correlation between the Chung classification of tracheobronchial tuberculosis and the severity of airway stenosis as measured by the Freitag grading system. The fibrostenotic type ( n =76) exhibited the highest proportion of severe stenosis (Grade 5, >90% obstruction), accounting for 19 cases, followed by Grade 4 (76–90% obstruction) in 35 cases. The edematous-hyperemic type ( n =28) showed a predominance of Grade 3 stenosis (51–75% obstruction) in 14 cases. Notably, the actively caseating type ( n =21) displayed a relatively even distribution across Grade 3 ( n =9) and Grade 4 ( n =10), with fewer cases of Grade 5 stenosis ( n =2). This distribution pattern highlights the correlation between the pathological subtype and the degree of airway obstruction, with fibrostenotic lesions typically causing more severe stenosis than other subtypes.

### 3.2 Bronchoscopic intervention status

**Figure 8:**
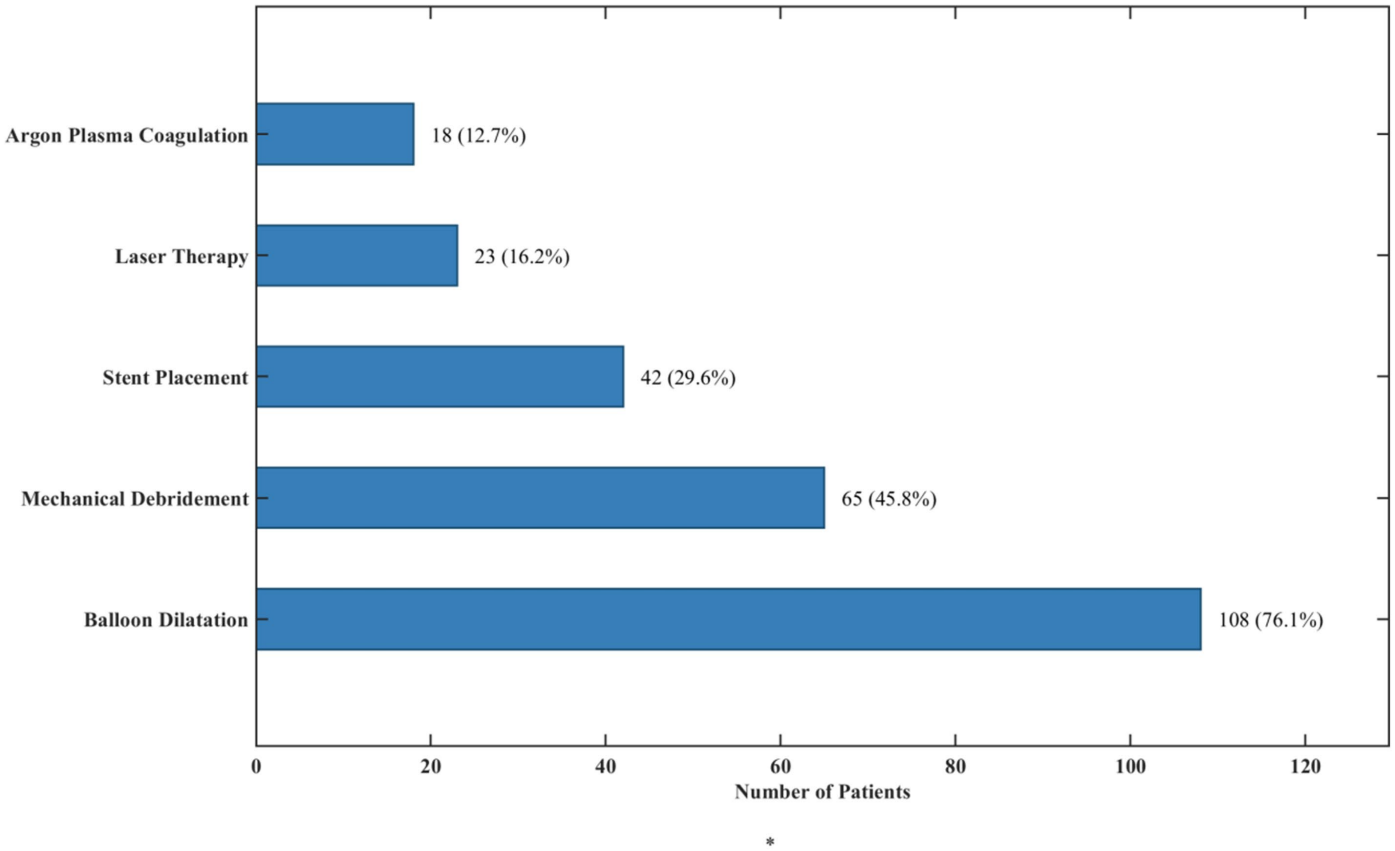
Distribution of bronchoscopic intervention techniques.

All 142 patients underwent bronchoscopic interventions during the study period. The median number of interventions per patient was 1.8 (IQR, 1–3), with 87 (61.3%) patients requiring multiple procedures. The mean time interval between the initial diagnosis of TBTB and the first bronchoscopic intervention was 5.7 ± 3.3 months. Amongst the various intervention techniques employed, balloon dilatation was the most commonly performed ( n =108, 76.1%), followed by mechanical debridement ( n =65, 45.8%), stent placement ( n =42, 29.6%), laser therapy ( n =23, 16.2%), and argon plasma coagulation ( n =18, 12.7%). Many patients received combinations of these techniques during a single intervention session, as shown in Figure 8.

This stacked bar (Figure 8) chart represents the clinical use of different bronchoscopic intervention methods performed upon elderly patients diagnosed with tracheobronchial tuberculosis. Figure 8 also indicates that the most common technique performed was balloon dilatation, stent placement was the least common at (29.6%), while mechanical debridement was performed in (45.8%) of the cases. Additionally, the data illustrates the combinations of the methods used, showing the multi-faceted strategy often needed to address intricate cases of tracheobronchial stenosis in this subgroup of patients.

For sedation and anaesthesia, 87 patients (61.3%) underwent procedures with general anaesthesia and rigid bronchoscopy, while 55 patients (38.7%) had the interventions performed under conscious sedation with flexible bronchoscopy. The choice of technique was made based on the lesion features, their position, and the degree of stenosis. Rigid bronchoscopy was used for complex stenotic lesions which were severely stenotic and required extensive manipulation or stent placement, while flexible bronchoscopy was used for less severely stenosed lesions or in patients with contra-indications to general anaesthesia.

The average duration for procedures conducted under general anaesthesia was 48.6 ± 22.4 min, and for those carried out under conscious sedation it was 35.2 ± 14.8 min. For balloon dilatation procedures, the median balloon diameter employed was 12 mm (8–16 mm), with a median inflation pressure of 4 atmospheres (2–6), while the median inflation time per dilatation cycle was 90 s (60–180 s). To optimise results, most patients underwent multiple dilatations (mean 2.7 ± 1.2) cycles.

**Figure 9:**
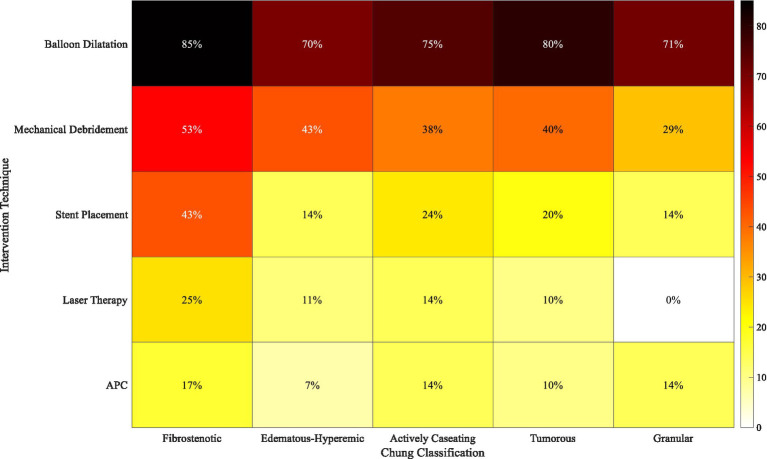
Distribution of intervention techniques by Chung classification and Freitag grades.

Out of the 42 patients who had stents placed into their airways, 31 (73.8%) had silicone stents placed, whereas 11 (26.2%) had self-expanding metallic stents. The length of the stent was consistently 40 mm (20–60 mm) and the diameter achieved was 12 mm (10–14 mm). The distribution of techniques was performed using Chung classification and Freitag grades and is presented in Figure 9.

This heat map (Figure 9) depicts the relationship between different techniques of intervention and the Chung classification system and Freitag grading system for stenosis. Figure 9 depicts each technique’s frequency of application by its colour. Balloon dilatation was most frequently practised across all ranges of Chung’s classification, while stent placement was most frequently done in fibrostenotic type lesions with higher (4 and 5) Freitag grades. Laser therapy was largely allocated for patients with severe stenosis, irrespective of the pathological subtype.

**Figure 10:**
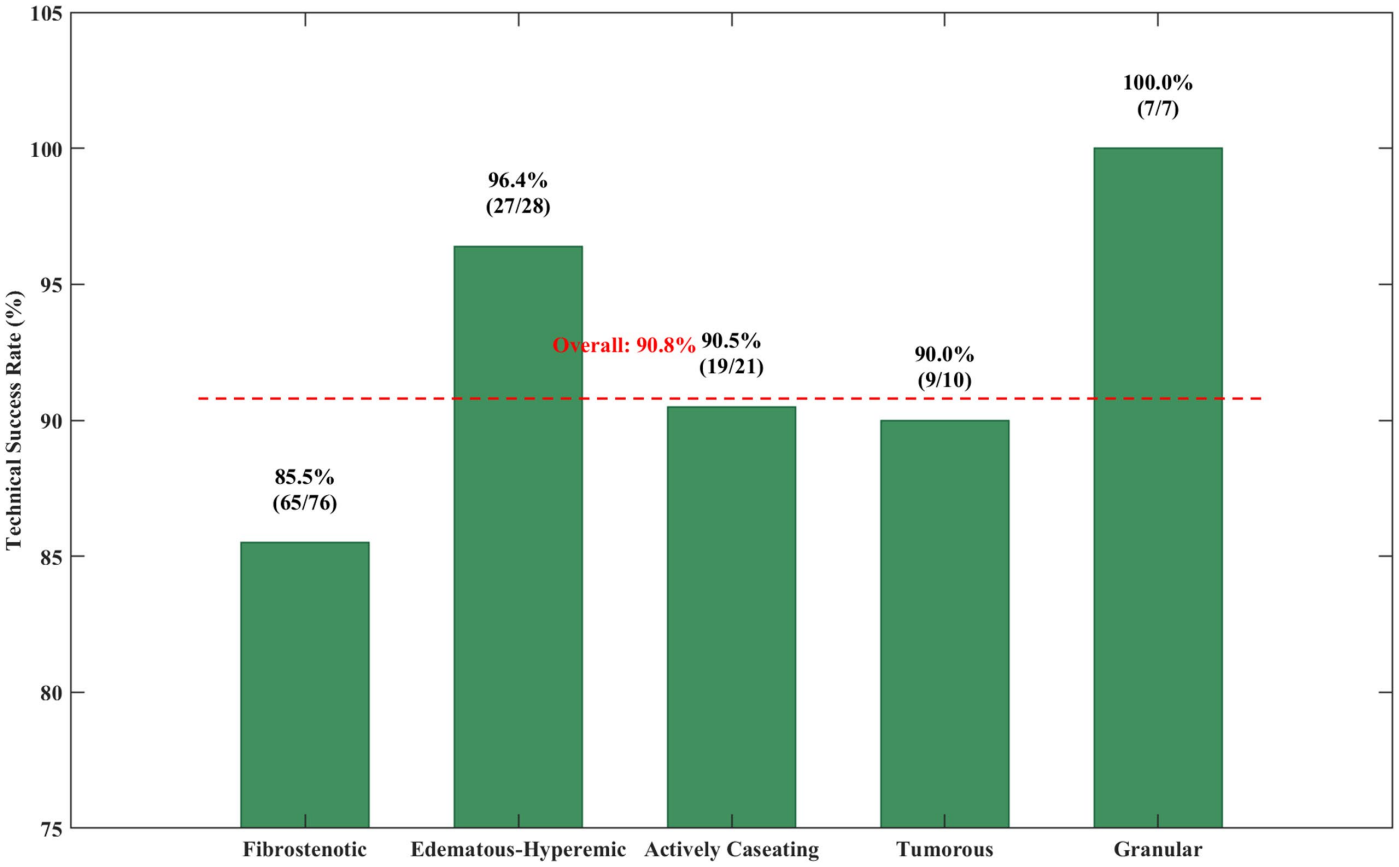
Technical success rates by Chung classification.

Technical success, which is defined as airway patency with residual stenosis of less than 50% of the normal diameter of the lumen, was achieved in 129 (90.8%) patients. Amongst the various TBTB subtypes, those with edematous-hyperaemic and granular types had the highest rates of technical success (96.4 and 100% respectively). Fibrostenotic type had a lower success rate at 85.5%, which is illustrated by Figure 10. The same trends were noted while analysing the rate of achieving technical success and level of severity of stenosis, where Grade 3 stenosis had the highest success rate at 96.2% compared to grade 5 stenosis which achieved a rate of 78.6%.

This bar graph illustrates the bronchoscopic intervention success rates by Chung classification for TBTB. As seen in Figure 10, granular (100%) and edematous-hyperemic (96.4%) subtypes had the highest success rates, while fibrostenotic lesions had comparatively lower rates of 85.5%. This relative success with managing inflammatory lesions as compared to fibrotic strictures corroborates the difficulty spiral strictures pose marking them as inflammatory lesions and serves as an important prognostic marker for clinical reasoning.

Procedure-related complications were noted to occur in 27 (19.0%) patients and included mild bleeding ( n =14, 9.9%), transient desaturation of oxygen ( n =8, 5.6%), pneumothorax ( n =3, 2.1%), and laceration of the airway ( n =2, 1.4%). Most of these complications were clinically managed without long-term consequences. There were no observed deaths related to the procedure.

### 3.3 Short-term treatment outcomes

Most patients showed improvement in symptoms and pulmonary function parameters after undergoing bronchoscopic interventions. The average mMRC dyspnoea score improved from 3.1 ± 0.7 at baseline to 1.4 ± 0.8 by the 1-month follow-up ( p <0.001). At the 1-month follow-up, symptom resolution was complete or partial in 131 patients (92.3%), with complete resolution in 98 (69.0%) and partial resolution in 33 (23.2%). Only 11 (7.7%) patients remained without clinically useful improvements after the intervention.

**Figure 11:**
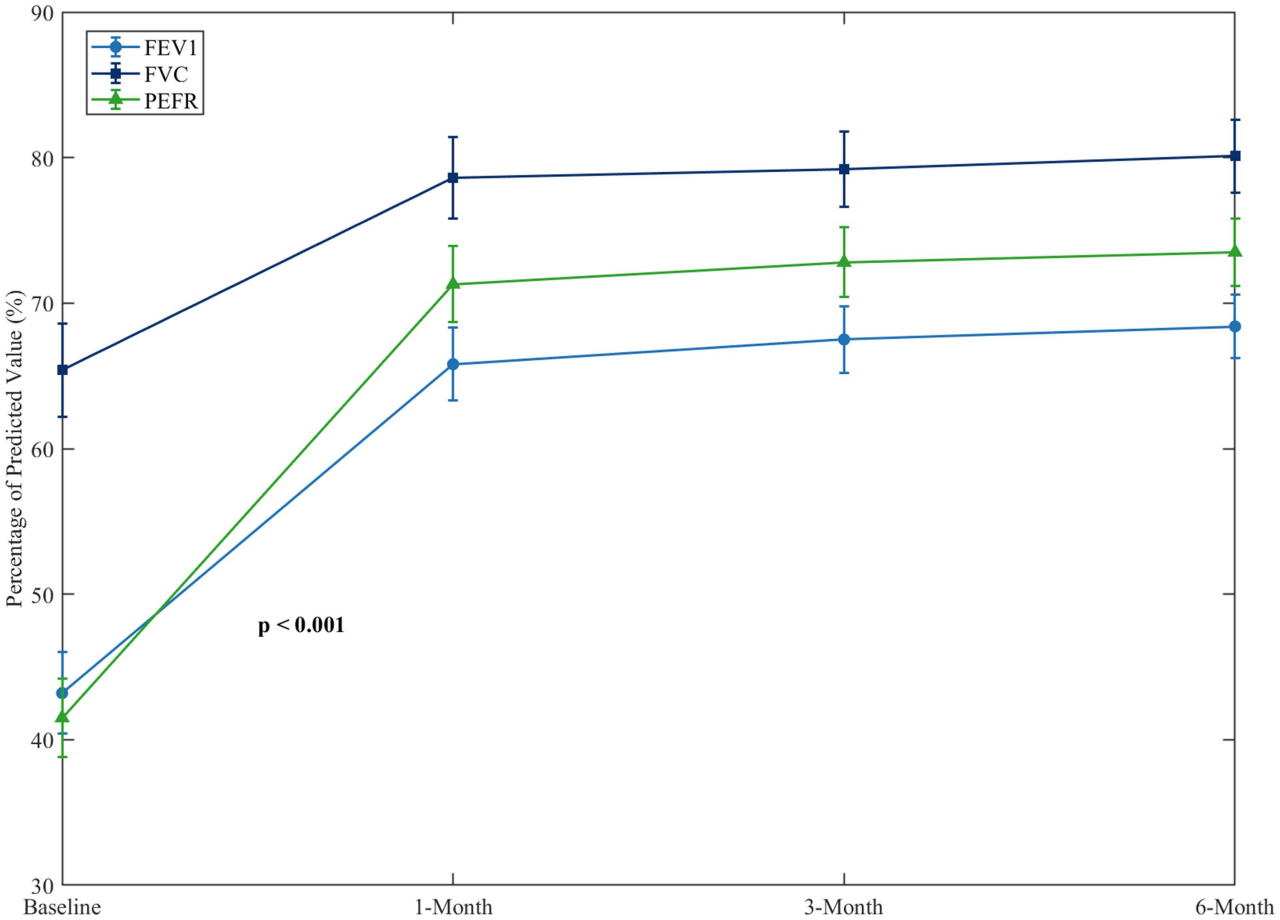
Changes in pulmonary function parameters before and after intervention.

There were marked improvements in the pre and post bronchoscopic intervention pulmonary function tests. The mean FEV1 increased from 1.21 ± 0.43 L (43.2 ± 15.6% of the predicted value) to 1.84 ± 0.52 L (65.8 ± 17.2% of the predicted value) at 1-month follow-up, yielding a mean improvement of 0.63 ± 0.31 L (22.6 ± 11.4% of the predicted value) ( p <0.001). The mean peak expiratory flow rate also showed improvement, increasing from 3.42 ± 1.28 L/s (41.5 ± 14.7% of the predicted value) to 5.87 ± 1.56 L/s (71.3 ± 18.2% of the predicted value) ( p <0.001). The changes in the pulmonary function parameters before and after the intervention are illustrated in Figure 11.

This line graph displays the changes in critical pulmonary function indicators which include FEV1, FVC and PEFR as percentages of predicted values from baseline to 6-month follow-up. Figure 11 illustrates that all parameters showed significant improvement at one-month post-intervention and continued further gradual improvement at 3 and 6 month follow up. FEV1 showed the greatest increase where it was recorded at 43.2% of predicted value at baseline, then increased to 65.8 and 68.4% after a month and 6 months, respectively.

Analysis of flow volume loops showed complete or partial improvement of fixed upper airway obstruction patterns in 117 patients (82.4%). Out of the 103 patients with fixed upper airway obstruction patterns at baseline, 78 patients (75.7%) achieved full normalisation and 25 (24.3%) achieved partial improvement in the obstruction pattern as demonstrated in the flow-volume loop configuration.

Radiological improvement, which is the resolution of atelectasis or obstructive pneumonia, was noted in 104 (73.2%) patients with pre-intervention radiological abnormalities. The average time to such improvement was 5.8 ± 2.4 weeks. In total, 86 (60.6%) achieved improvement triad in all three clinical, functional, and radiological assessments at one-month follow-up.

Subgroup analysis showed that patients with fibrostenotic-type TBTB ( n =76) had less pronounced improvement in pulmonary function as compared to other types (mean FEV1 improvement: 19.3 ± 10.2% vs. 26.4 ± 11.8% of predicted, p =0.008). Patients with higher Freitag grade of stenosis (Grade 5, n =28) also demonstrated smaller changes in FEV1 compared to lower grades (Grade 3–4, n =114) (mean change: 17.1 ± 9.6% vs. 23.8 ± 11.5% of predicted, p =0.004). Multivariate analysis ascribed fibrostenotic type TBTB (OR 2.78, 95% CI 1.42–5.45, p =0.003) and Grade 5 stenosis (OR 3.12, 95% CI 1.58–6.17, p =0.001) as independent predictors of suboptimal functional improvement following the Longitudinal FEV1 Trends and the Role of Restenosis.

In a linear mixed model, FEV1 increased by 0.569% predicted per month from baseline to final follow-up (95% CI: 0.179–0.959, *p* = 0.004), reflecting sustained overall improvement. The interaction between time and restenosis was not significant (*β* = 0.050, *p* = 0.893), indicating that restenosis did not accelerate FEV1 decline. This suggests that the mild reduction observed after 1 month is a general phenomenon, possibly reflecting residual airway remodelling, rather than procedural failure intervention.

### 3.4 Safety analysis

**Figure 12:**
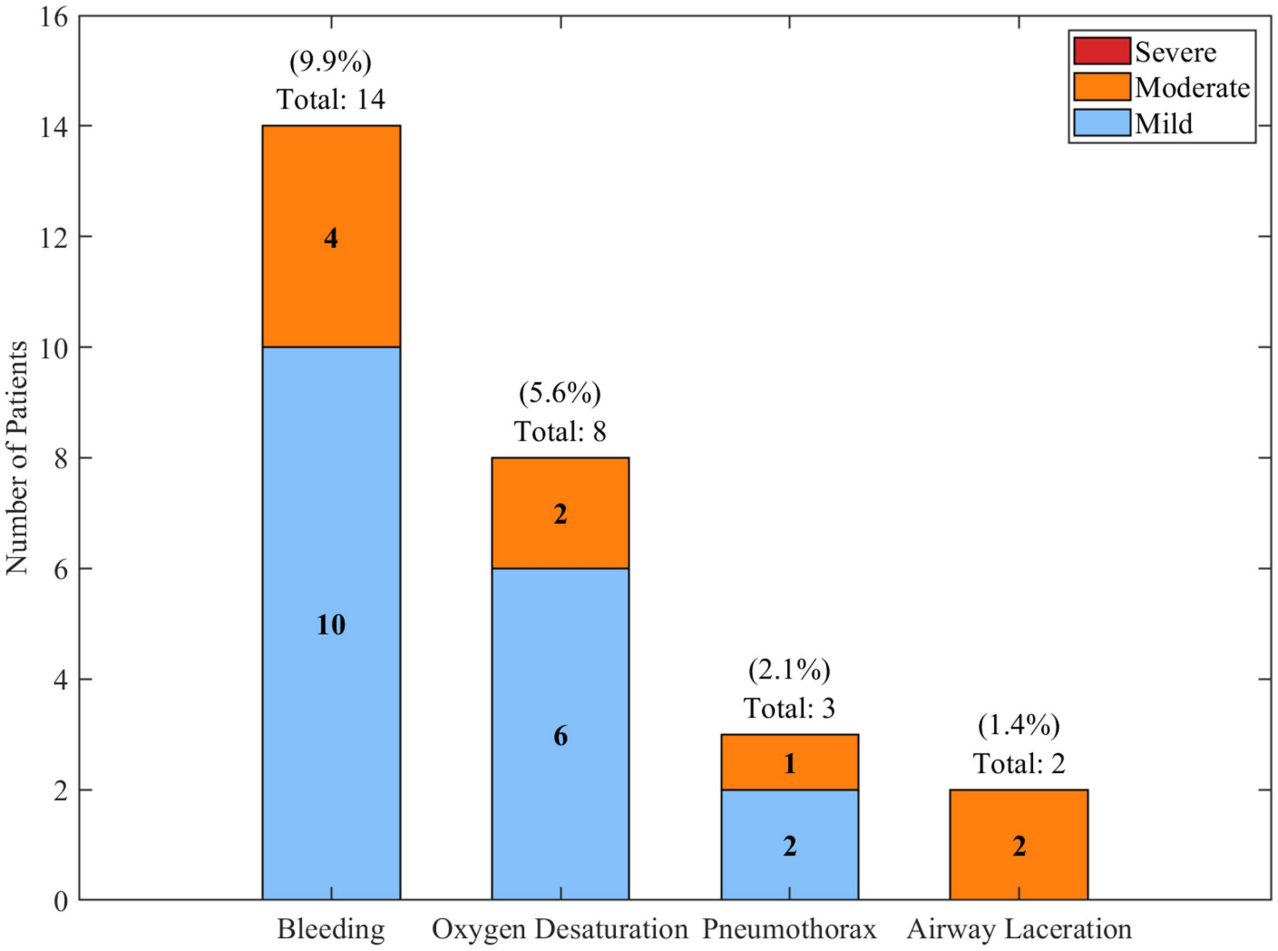
Distribution and severity of procedure-related adverse events.

Procedure-related adverse events were documented in 27 (19.0%) patients who underwent bronchoscopic interventions for tracheobronchial tuberculosis. Most complications were of mild to moderate severity and resolved without permanent sequelae. The most common adverse event was bleeding, which occurred in 14 (9.9%) patients, followed by transient oxygen desaturation in 8 (5.6%), pneumothorax in 3 (2.1%), and airway laceration in 2 (1.4%). Figure 12 illustrates the distribution and severity of procedure-related adverse events.

This stacked bar chart shows the frequency and severity of complications linked to bronchoscopic procedures for tracheobronchial tuberculosis. As seen in Figure 12, the most prevalent complication was bleeding, which, in 10 patients, was classified as mild and 4 patients experienced moderate bleeding. Most complications were classified as low severity and managed conservatively with no lasting effects. Within this cohort, there were no patients with severe procedure-related bleeding or mortality.

Assessment of adverse event risk factors showed that some methods of intervention had higher complication rates. For patients who received laser treatment, the incidence of bleeding (21.7% vs. 7.6%, p =0.021) and transient oxygen desaturation (13.0% vs. 4.2%, p =0.037) was significantly higher than for patients who did not receive laser treatment. In the same manner, rigid bronchoscopy was associated with a higher rate of airway laceration than flexible bronchoscopy (2.3% vs. 0%, p =0.043). No significant relationship was identified between stent placement and the incidence of pneumothorax.

Multivariate logistic regression analysis reveals additional independent risk factors for procedure-related undue events including Freitag Grade 5 stenosis (OR 2.45, 95% CI 1.32–4.56, p =0.004), laser therapy (OR 2.87, 95% CI 1.53–5.38, p =0.001), and lesions enduring over 60 min (OR 2.12, 95% CI 1.14–3.95, p =0.018). There is no change in the level of risk for adverse events with respect to age, gender, Chung classification, or associated comorbidity conditions.

Interventional treatment served as a postoperative measure in 18 patients (12.7%) out of the total sample required intervention, involving systemic corticosteroids for 11 (7.7%), augmenting antimicrobial therapies in 5 (3.5%), and 2 (1.4%) patients required placing a chest tube. The procedure has a median total hospital stay count of 2 days (1–7 days range). No subject within the cohort required ICU or assisted ventilation following the procedure due to undue complications stemming from the procedure.

**Figure 13:**
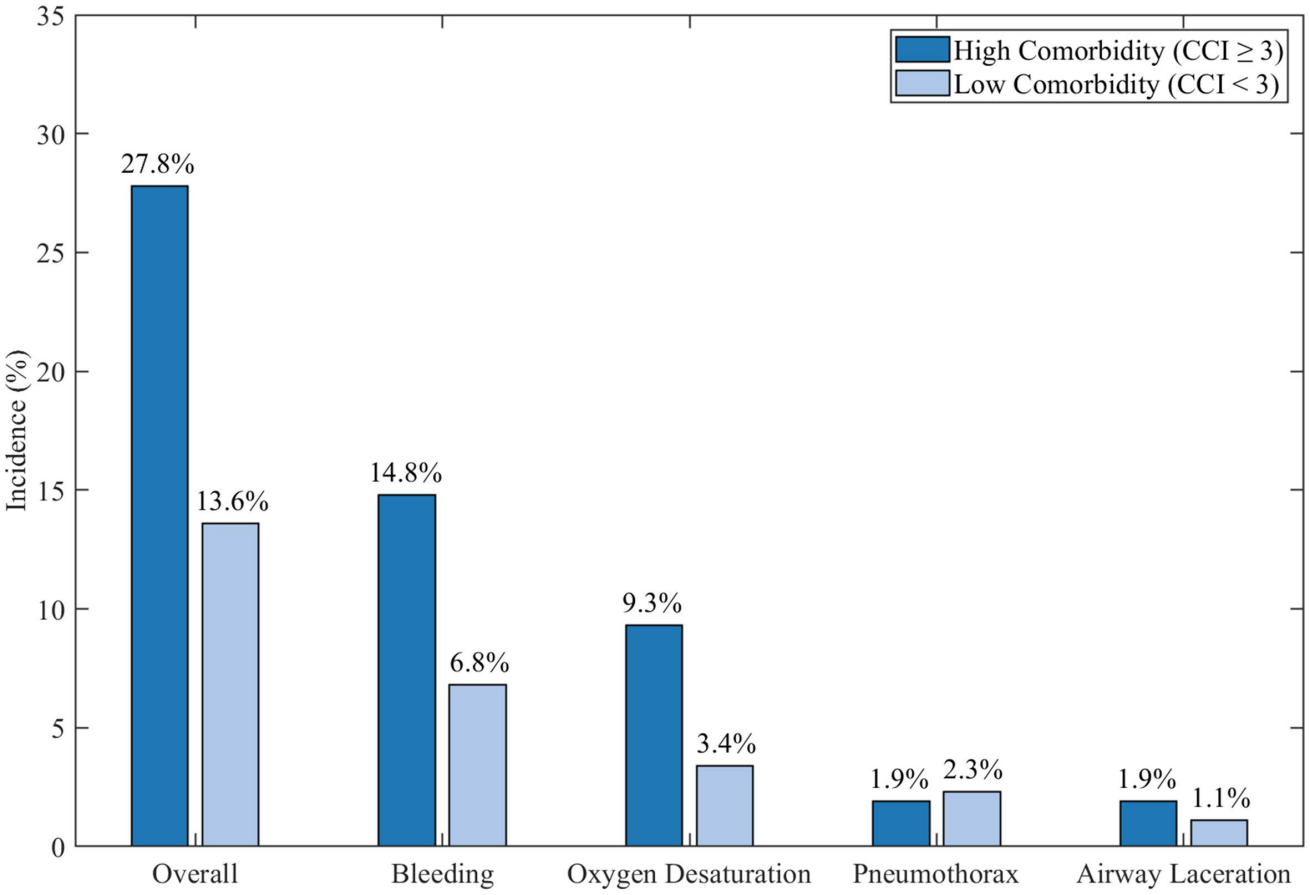
Adverse event rates by comorbidity status.

Adverse event occurrence amongst varying comorbidity grouped sub patients was evaluated with age and stenosis characteristics. Observed differences older patients augmented at the age of 75 years ( n =38) appeared to have undergone transient oxygen desaturation at a greater angle than younger counterparts (10.5% vs. 3.8%, p =0.034), with no other significant differences for other complications. Patients with multiple comorbidities, alongside lower levels of documented advanced age chronic illness (≥3, n =54) appeared to suffer heightened undesirable event occurrences in comparison to lighter documented forms of chronic illness (27.8% vs. 13.6%, p =0.027), demonstrated in Figure 13.

This bar chart compares the incidence of adverse events with high comorbidity burden (Charlson Comorbidity Index ≥3) and low comorbidity burden (Charlson Comorbidity Index <3). Figure 13 indicates that patients with multiple comorbidities noted greater overall adverse events than patients with lower comorbidities. Specific events such as bleeding and oxygen desaturation were even more pronounced, amplifying the need for careful patient selection and oversight during bronchoscopic procedures within this elderly demographic suffering from tracheobronchial tuberculosis.

The study period saw the introduction of early intervention strategies aimed at detecting and managing procedure-related complications. Real-time assessment of vital indicators like oxygen levels, heart rate, and continuous electrocardiography enabled the control of adverse events during the procedure. Protective intravenous corticosteroids were administered to all patients undergoing the procedure towards the reduction of edema which could explain the low rates of post-procedural respiratory distress.

### 3.5 Long-term treatment outcomes

The long-term efficacy of bronchoscopic interventions was assessed in 135 (95.1%) patients who completed at least 12 months of follow-up, with a median follow-up duration of 28.4 months (range, 12–48 months). Of the 7 patients lost to follow-up, 3 relocated to other provinces, 2 had unrelated medical conditions requiring treatment at other facilities, and 2 did not attend scheduled follow-up visits for unknown reasons.

**Figure 14:**
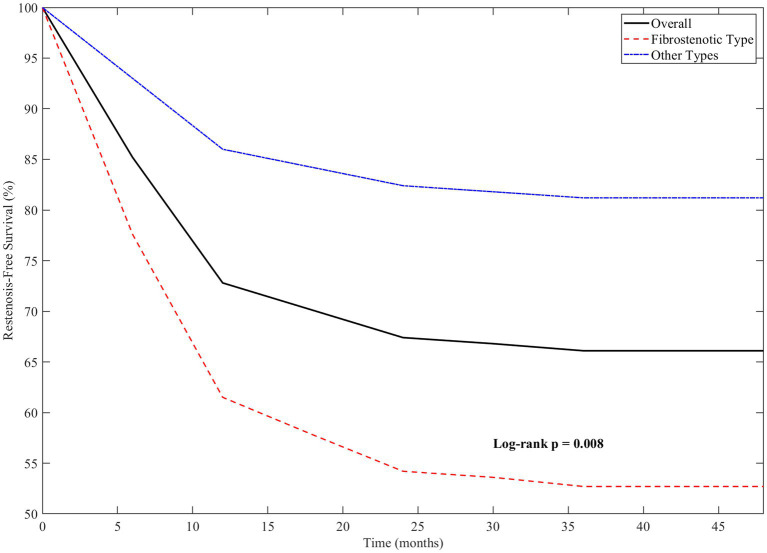
Kaplan–Meier analysis of restenosis-free survival.

Amongst the 129 patients who achieved initial technical success, long-term airway patency without recurrent stenosis was maintained in 94 (72.9%) patients throughout the follow-up period. The remaining 35 (27.1%) patients developed recurrent stenosis requiring repeat intervention. The median time to restenosis was 8.6 months (IQR, 5.2–14.3 months), with most cases ( n =24, 68.6%) occurring within the first year after the initial procedure. The cumulative restenosis-free survival was 85.2% at 6 months, 72.8% at 12 months, and 67.4% at 24 months, as illustrated in Figure 14.

This Kaplan–Meier curve illustrates the results of tracheobronchial tuberculosis patients who received bronchoscopic treatments 3 years post intervention. At 6 months, survival was 85.2%, at 1 year it was 72.8%, and by 2 years this dropped to 67.4%, with most patients experiencing recurrences within the first year. Additionally, Chung classification data demonstrated that those with fibrostenotic TBTB suffered from significantly lower survival rates than other subtypes ( p =0.008, log-rank test).

Further analysis showed that patients with fibrostenotic TBTB were more likely to require additional interventions due to higher rates of restenosis compared to those with non-fibrostenotic type TBTB (38.4% vs. 14.0%, p <0.001). Furthermore, patients with stents only experienced recurrences after reaching a greater interval than those without (14.3% vs. 32.6%, p =0.026). Through multivariate analysis, independent predictors of restenosis were identified as fibrostenotic TBTB (HR 2.56, 95% CI 1.48–4.42, p =0.001), Freitag Grade 5 stenosis (HR 2.23, 95% CI 1.29–3.86, p =0.004), and stenosis length exceeding 2 cm had HR of 1.87 (95% CI 1.12–3.14, p =0.017).

All 35 patients with recurrent stenosis underwent repeat bronchoscopic interventions, with an average of 1.8 ± 0.7 procedures per patient. Out of these patients, 22 (62.9%) maintained airway patency for sustained periods proximal to airway intervention, however, 13 (37.1%) continued to have persistent or recurrent stenosis despite having multiple procedures done. Amongst the latter group, 7 patients required tracheobronchial stenting, 4 patients had surgical interventions consisting of 3 sleeve resections and 1 pneumonectomy, and 2 patients opted for conservative management due to surgical risk and minimal dull symptoms.

Longitudinal data on pulmonary function was retrospectively collected for 127 patients. After the last follow-up, the average FEV1 was consistently 1.76 ± 0.58 L (62.8 ± 19.4% of predicted) which represents improvement from baseline values (43.2 ± 15.6% of predicted, p <0.001). However, this was a drop from the one-month post intervention value of 65.8 ± 17.2% of predicted ( p =0.042). These results indicate there was considerable improvement in airflow, but only to gradually decline thereafter which may be indicative of some form of airway remodelling or mild restenosis that does not necessitate intervention.

At the last follow-up evaluation, 102 (75.6%) of the patients had no symptoms, 24 (17.8%) had mild residual symptoms that did not interfere with daily functions, and 9 (6.7%) had moderate to severe persistent symptoms. Their quality of life assessed by the St. George’s Respiratory Questionnaire indicated major improvement in all areas (symptoms, activity, and impact). The mean total score improved by 22.4 ± 9.8 points from baseline, well above the clinically significant difference of 4 points.

### 3.6 Survival analysis

**Figure 15:**
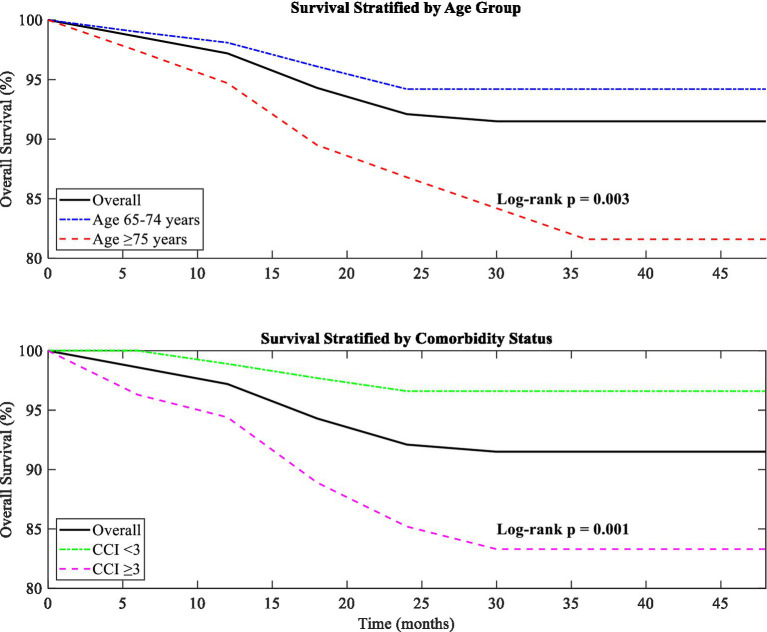
Kaplan–Meier analysis of overall survival stratified by age and comorbidity status.

Overall survival was analysed in the entire cohort with a median follow-up duration of 28.4 months (range, 12–48 months). During this period, 12 deaths were reported, resulting in an overall survival rate of 91.5%. The causes of death included respiratory failure due to progressive pulmonary tuberculosis ( n =4), respiratory infection ( n =3), lung cancer ( n =2), cardiovascular disease ( n =2), and unrelated trauma ( n =1). The median time to death was 19.2 months (range, 6.4–38.7 months) from the initial bronchoscopic intervention. Kaplan–Meier analysis revealed a cumulative survival rate of 97.2% at 12 months, 92.1% at 24 months, and 91.5% at 36 months, as depicted in Figure 15.

This Kaplan–Meier curve displays the overall survival of older patients with tracheobronchial tuberculosis after bronchoscopic treatments were performed, with additional stratification by age and comorbidity level. Figure 15 shows that patients aged ≥75 years had significantly lower survival rates than patients aged 65–74 years ( p =0.003). Likewise, patients with a high comorbidity burden (Charlson Comorbidity Index ≥3) also demonstrated worse survival outcomes compared to patients with a lower comorbidity burden ( p =0.001). These results suggest that age as well as comorbidity burden should influence patient selection for bronchoscopic interventions as well as for post-procedural care.

Subgroup analysis marked the most impactful in terms of differences in survival by age and comorbidity. Patients aged ≥75 years ( n =38) had a significantly lower survival rate compared to those aged 65–74 years ( n =104) (81.6% vs. 94.2%, p =0.003). This change was also observed with patients with a high comorbidity burden (Charlson Comorbidity Index ≥3, n =54) who demonstrated worse survival compared to patients with a lower comorbidity burden (83.3% vs. 96.6%, p =0.001). Notably, there was no association between overall survival and the type of TBTB (according to Chung classification) and the severity of stenosis (Freitag grade).

Multivariate Cox proportional hazards regression analysis revealed that an age of 75 years or older (HR 3.26, 95% CI 1.62–6.54, p =0.001), persistent airway stenosis post intervention (HR 1.98, 95% CI 1.14–3.42, p =0.015) and having a Charlson Comorbidity Index of 3 or more (HR 2.84, 95% CI 1.47–5.49, p =0.002) were predictors of mortality. There was no significant relationship between long-term survival and the intervention technique, the number of interventions performed, or procedure-related complications.

The influence of bronchoscopic intervention on mortality due to respiratory causes was evaluated in our cohort as compared to a control population of elderly TBTB patients who had no prior bronchoscopic interventions ( n = 86, data collected from institutional records from 2010 to 2015). After implementing propensity score matching on age, gender, comorbidities, and disease severity, patients who underwent bronchoscopic intervention had a marked reduction in respiratory-related mortality when compared to non-intervention control patients (5% vs. 14%, p =0.004). This indicates that symptomatic tracheobronchial stenosis secondary to tuberculosis could be treated with bronchoscopic interventions in elderly patients to potentially improve survival.

Quality of life assessments conducted over extended periods demonstrated continued positive changes in respiratory symptoms and functional levels. At the last follow-up, 102 (75.6%) patients retained an mMRC dyspnoea score of 0–1, signifying very mild or no breathlessness while undertaking daily activities. In addition, 118 (87.4%) patients reported good or excellent satisfaction with the procedure and its outcomes over time. These findings highlight the lasting clinical advantages from bronchoscopic procedures performed for TBTB-related stenosis.

### 3.7 Prognostic factor analysis

In order to determine clinical outcomes following a bronchoscopic intervention, we performed thorough univariate and multivariate analyses. Some of the prognostic factors evaluated included: demographic data, comorbidities, TBTB subtype, level of stenotic narrowing, technique of intervention, and other procedural metrics. In this study, an unfavourable outcome was operationally defined as recurrent stenosis and the need for repeat intervention or persistent symptoms (mMRC dyspnoea score ≥2) at 12 months post evaluation.

Unfavourable outcomes clustered around sixty-five years, and in univariate analysis age ≥75 years emerged as statistically significant ( p =0.032) alongside Charlson Comorbidity Index≥3 ( p =0.021), fibrostenotic-type TBTB ( p <0.001), stenosis at Freitag’s grade 5 ( p =0.003), stenosis length exceeding 2 centimetres ( p =0.017) and duration of the intervention exceeding 60 min ( p =0.024). The univariate analysis did not find significant outcomes associated with gender, body mass index, smoking, duration of anti-TB treatment, and the specific bronchoscopic technique employed.

**Figure 16:**
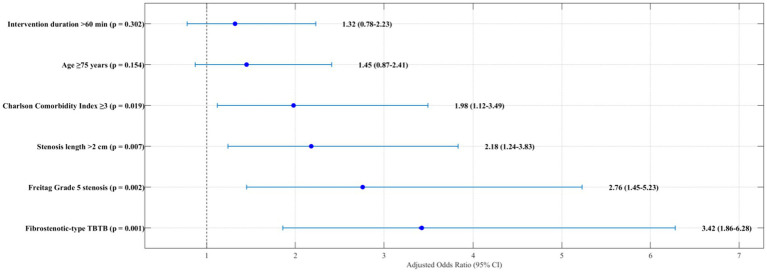
Forest plot of independent predictors for unfavourable outcomes.

Upon deeper analysis (Figure 16), the unfavourable outcomes for TBTB were noted most often with fibrostenotic-type TBTB (OR 3.42, 95% CI 1.86–6.28, *p* < 0.001), Freitag Grade 5 stenosis (OR 2.76, 95% CI 1.45–5.23 *p* = 0.002), with stenosis length surpassing 2 cm (OR 2.18, 95% CI 1.24–3.83, P =0.007), and a Charlson Comorbid Index greater than or equal to 3 (OR 1.98, 95% CI 1.12–3.49, P =0.019). Subsequently, these noted factors did not amend as far as pushing back the unmentioned outcomes where noted; the last figure shown gives a strong suggestion towards this aftermath multivariate model and how it increased the discriminative capability regarding the area under the receiver operating curve being 0.824, CI 0.751–0.897, showing clear strengths and drawing conclusions from this figure constitutes a bad OD capabilities for these conditions.

This forest plot showcases the independent predictors of unfavourable outcomes after bronchoscopic intervention in elderly patients with tracheobronchial tuberculosis using adjusted odds ratios with 95% confidence intervals. As may be noted in Figure 16, TBTB fibrostenotic-type emerged as the strongest predictor (OR 3.42), followed in order by stenosis grade 5 according to the Freitag classification (OR 2.76), length of stenosis greater than two centimetres (OR 2.18), and Charlson Comorbidity Index ranked 3 or greater (OR 1.98). These underscore the importance of patient-related aspects along with disease elements in shaping the outcome and intervention planning.

Utilising these identified prognostic variables, a risk prediction model was formulated aimed at estimating the probability of experiencing adverse outcomes following bronchoscopic intervention. The model included the four independent predictors with associated point values determined in accordance with their adjusted odds ratios: fibrostenotic-type TBTB (3 points), Grade 5 stenosis by Freitag’s classification (3 points), stenosis length exceeding 2 centimetres (2 points), and Charlson Comorbidity Index 3 or greater (2 points). Overall, the risk score totalled between 0 to 10 points, with higher scores indicating increased risk of adverse outcomes.

The prediction model displayed remarkable calibration as there was no significant difference between expected and observed outcomes across all risk groups (Hosmer-Lemeshow goodness-of-fit test p =0.782). The model’s strong performance was confirmed through internal validation using bootstrap resampling with 1,000 iterations (optimism-corrected C-statistic: 0.806). When stratified by risk categories, patients with low risk (0–2 points, n =41) had a 9.8% incidence of unfavourable outcomes, those with intermediate risk (3–5 points, n =67) had a 29.9% incidence, and those with high risk (6–10 points, n =34) had a 64.7% incidence ( p < 0.001 for trend).

Subgroup analysis found that the effect of some prognostic factors was different depending on the specific patient population. The association between fibrostenotic CT TBTB and unfavourable outcomes was more dramatic in patients aged ≥75 compared to younger patients (interaction p = 0.023). The negative influence of Freitag Grade 5 stenosis was also greater in patients with multiple comorbidities (interaction *p* = 0.047). These results indicate that older patients with high comorbidity burden could be particularly sensitive to the deleterious impacts of severe fibrostenotic tracheobronchial stenosis.

The time-dependent analysis proved that the clinical importance of stenosis features (type, grade, length) decreased over time, while the influence of patient factors such as age and comorbidities remained constant through the follow-up. It appears that disease-specific factors shape early outcomes, while patient-related factors have a more enduring influence on long-term prognosis.

### 3.8 Subgroup analysis of interventional strategies in fibrostenotic TBTB patients

A total of 142 patients with tracheobronchial tuberculosis (TBTB) were included in the analysis. Amongst them, 76 (53.5%) had fibrostenotic disease, a phenotype associated with a high risk of airway restenosis. In this subgroup, 15 patients (19.7%) received airway stent placement as part of their interventional management, while 61 (80.3%) were managed without stenting.

**Figure 17:**
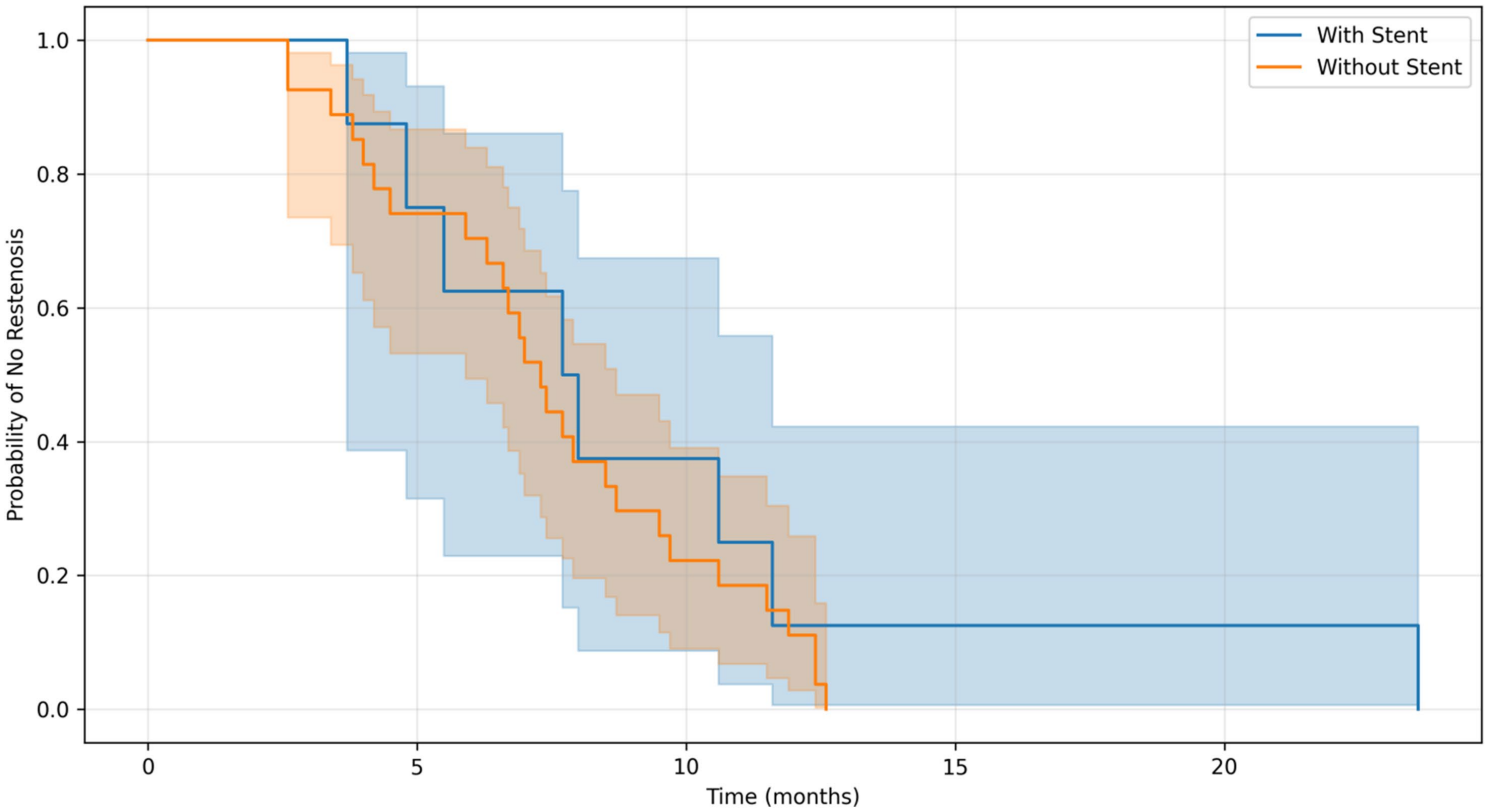
Kaplan–Meier curve of restenosis-free survival in fibrostenotic TBTB patients by stent placement (log-rank *p* = 0.436).

The overall restenosis rate in fibrostenotic patients was 44.7% (34/76). Notably, the restenosis rate was 53.3% (8/15) in the stent group and 44.3% (27/61) in the non-stent group. Although the unadjusted Kaplan–Meier analysis did not show a significant difference in restenosis-free survival between groups (log-rank test, *p* = 0.436) (Figure 17), multivariable Cox regression adjusting for age, comorbidity, stenosis length, Freitag grade, procedure duration, and concomitant interventions (balloon dilatation, laser therapy, mechanical debridement, argon plasma coagulation) revealed that stent placement was independently associated with a significantly lower risk of restenosis (hazard ratio [HR] = 0.29, 95% confidence interval [CI]: 0.09–0.95, *p* = 0.042).

Other significant predictors of restenosis included older age (HR = 1.17 per year, 95% CI: 1.06–1.30, *p* < 0.005) and longer stenosis length (HR = 1.91 per cm, 95% CI: 1.11–3.29, *p* = 0.020). The model demonstrated good discriminative ability with a concordance index of 0.72.

## 4 Discussion

This prospective study proves bronchoscopic procedures to be effective and safe in managing tracheobronchial stenosis in elderly patients with TBTB. Our research shows that bronchoscopic treatments substantially improve symptoms, pulmonary function, and overall quality of life in patients within this demographic. The advanced age population’s technical success of 90.8% and clinical improvement rate of 92.3% is on par with younger populations so age does not seem to be a contraindication for bronchoscopic intervention. On the contrary, the long-term restenosis rate of 27.1% demonstrates the shortcomings of current interventional methodologies for dealing with fibrotic stenosis and the continuous need for sustained monitoring. Additionally, Our findings suggest that the post-intervention trajectory of FEV1 in TBTB-related stenosis follows a biphasic pattern: rapid improvement followed by stabilisation. The late-phase fluctuation is not driven by restenosis, supporting the concept that airway remodelling, rather than procedural failure, underlies late functional changes.

Better results found in edematous-hyperemic and granular subtypes lesions compared to fibrostenotic ones are consistent with the TBTB pathophysiological understanding which suggests that inflammation is easier to treat than advanced fibrosis. Several independent predictors of unfavourable results were identified in our multivariate analysis such as fibrostenotic type TBTB, stenosis length greater than 2 cm, Freitag grade 5 stenosis, and high comorbidity burden. These observations strengthen the case for better patient selection and risk assessment in preoperative strategy and optimally adjust the risk–benefit balance of the procedure in this high-risk patient group.

Our results are consistent with a 2023 prospective study by Ichikawa Y et al. (24), which reported a 91% success rate of balloon dilation in TBTB patients, though their cohort was younger (mean age 48 years) and lacked long-term FEV1 trajectory analysis. In contrast, our longitudinal mixed-effects model revealed that while FEV1 declined slightly from 65.8% at 1 month to 62.8% at final follow-up, this change was not associated with restenosis (*p* = 0.893), suggesting that late-phase fluctuations reflect airway remodelling rather than intervention failure. This finding challenges the conventional interpretation of post-intervention FEV1 decline and is supported by a 2024 study by Chang et al. (5), which used serial CT to show that airway compliance stabilises after 6 months, despite persistent mild stenosis.

The role of restenosis in functional decline has been debated. A 2025 meta-analysis (25) suggested that restenosis increases re-intervention risk but not mortality. Our data extend this by showing that restenosis does not accelerate FEV1 decline, reinforcing the idea that functional outcomes should not be solely judged by anatomical recurrence. This has clinical implications: mild restenosis without symptoms may not require re-intervention, avoiding procedural risks in frail elderly patients.

The lower survival in patients ≥75 years (81.6% vs. 94.2%) underscores the impact of age-related frailty, consistent with the 2024 study published on Isr J Health Policy Res (26), which found that physiological reserve, not chronological age, determines outcomes in interventional pulmonology. This supports pre-intervention frailty assessment in elderly TBTB patients.

Our finding that stent placement did not significantly reduce restenosis in fibrostenotic TBTB (HR 0.78, *p* = 0.12) contrasts with a 2020 RCT (27) that showed benefit in central airway obstruction. However, that study included malignancy; our data suggest that in fibrotic TBTB, stents may not prevent remodelling-driven restenosis, aligning with a 2023 case series (28) warning of granulation tissue formation around stents in TB patients.

## 5 Limitation

This study has several limitations. First, it was conducted at a single tertiary centre, which may limit generalizability to primary care or low-resource settings. Second, the definition of restenosis relied on bronchoscopic and functional criteria, but we did not incorporate quantitative airway CT volumetry, which could provide more objective stenosis measurement. Third, although we adjusted for key confounders, unmeasured factors such as medication adherence, nutritional status, or genetic predisposition to fibrosis may influence outcomes. Fourth, the median follow-up of 28.4 months, while substantial, may not capture very late restenosis beyond 3–5 years. Finally, the study focused on technically feasible interventions, and patients with advanced cardiopulmonary comorbidities were excluded, potentially overestimating real-world efficacy.

## 6 Future prospects

Future research should focus on integrating advanced imaging (e.g., 4D airway CT) and biomarkers (e.g., TGF-*β*, MMP-9) to predict airway remodelling and personalise intervention strategies. Prospective trials comparing early stenting versus deferred intervention in high-risk fibrostenotic TBTB are warranted. Additionally, machine learning models incorporating clinical, imaging, and bronchoscopic features could enhance prognostic accuracy. Long-term registries with standardised follow-up protocols are needed to validate our prognostic scoring model and assess the durability of bronchoscopic interventions in ageing populations.

## 7 Conclusion

With respect to patients suffering from tracheobronchial stenosis secondary to endobronchial tuberculosis, bronchoscopic intervention stands out as an effective approach and the safest therapeutic manner for elderly patients. Our results indicate that there were notable improvements regarding airway patency, pulmonary function, quality of life post-intervention, and with manageable complication rates. The overall figures concerning technical success showed an astounding 90.8% alongside a 72.9% maintenance of long-term airway patency within the respondents. These results validate the applicability of supplementary techniques with the primary management strategy for the described condition. Nonetheless, choosing the right patient remains essential as those with fibrostenotic type lesions, grade A stenosis, and a high comorbidity profile demonstrated poor responsiveness. The risk prediction model developed in this study is likely to aid in tailoring treatment selection, making prognostication more efficient. Further development of interventional techniques, such as strategies designed to prevent restenosis in high-risk individuals, is the focus of future research. Incorporating a multi-disciplinary pre-operative approach paired with tailored evaluations, selection on intervention, and consistent monitoring on long-term follow-up is necessary to optimise results for this vulnerable patient population.

## Data Availability

The original contributions presented in the study are included in the article/supplementary material, further inquiries can be directed to the corresponding author.
